# Is Emotional Allodynia in Fibromyalgia Reducible to Depression? A Preliminary Network Analysis in 149 Patients

**DOI:** 10.3390/medsci14060599

**Published:** 2026-09-23

**Authors:** Alberto Corriero, Mariateresa Giglio, Alfonso Pilolla, Filomena Galdini, Cinzia Di Venosa, Elisabetta Costantino, Daniela De Orsi, Giustino Varrassi, Paolo Trerotoli, Filomena Puntillo

**Affiliations:** 1 Pain and ICU Section, Department of Interdisciplinary Medicine, University of Bari Aldo Moro, Piazza G. Cesare 11, 70124 Bari, Italy; mariateresa.giglio@uniba.it (M.G.); alfonso.pilolla@gmail.com (A.P.); milena200392@gmail.com (F.G.); cinziadivenosa@gmail.com (C.D.V.); filomena.puntillo@uniba.it (F.P.); 2 Nutrimente Onlus, 20121 Milano, Italy; elisabettacostantinopsi@gmail.com; 3 Centro di Salute Mentale Area 5, ASL Bari, 70124 Bari, Italy; daniela.deorsi@asl.bari.it; 4 Paolo Procacci Foundation, 00193 Rome, Italy; giuvarr@gmail.com; 5 Statistics Section, Department of Interdisciplinary Medicine, University of Bari Aldo Moro, Piazza G. Cesare 11, 70124 Bari, Italy; paolo.trerotoli@uniba.it

**Keywords:** fibromyalgia, nociplastic pain, emotional allodynia, affective symptoms, emotional regulation, catastrophization, central nervous system sensitization, depression, anxiety, statistical models

## Abstract

**Background/Objectives:** Because affect is challenging to quantify in fibromyalgia, previous network analyses have primarily relied on symptom-severity measures, whereas emotion regulation and affective hypersensitivity have rarely been included. We examined where emotional allodynia, the tendency to respond with disproportionate distress to neutral or low-intensity interpersonal cues, sits in this network. **Methods:** Cross-sectional analysis of 149 consecutive outpatients with fibromyalgia (2016 American College of Rheumatology criteria; 91.9% women; mean age 57.5 years), a cohort that includes the participants of two previous reports. A regularized partial correlation network (EBICglasso: extended Bayesian information criterion graphical lasso; γ = 0.5, Spearman) was estimated over twelve nodes: emotional allodynia, pain catastrophizing, central sensitization, depression, state and trait anxiety, and six facets of emotion dysregulation. Accuracy was assessed with 5000 bootstrap replications. **Results:** The correlation of emotional allodynia with depression (ρ = 0.502) was shrunk to zero in the network and under every sensitivity analysis, and was near zero when the penalty was removed (ρ = −0.033). Its two strongest edges were with pain catastrophizing (0.190) and the Impulse facet of emotion dysregulation (0.184), non-zero in 98.9% and 99.6% of replications. It was among the less connected nodes, but, forming a community of one, all its connectivity crossed a domain boundary: 59.8% went to emotion regulation and 30.2% to pain, the largest share of any node outside either domain (77.0% and 48.1% of replications). Depression and anxiety received 10.0% (24.6% for catastrophizing). **Conclusions:** In this exploratory analysis, the connectivity of emotional allodynia was directed at emotion regulation and pain rather than at depression and anxiety, and emotional allodynia showed little residual association with depressive symptom severity once the other measures were held constant. It overlapped substantially with catastrophizing, and these data do not adjudicate their separability. These preliminary single-centre findings are hypothesis-generating and require replication.

## 1. Introduction

Fibromyalgia is the prototypical nociplastic pain condition, in which pain arises from altered nociception in the absence of identifiable tissue damage or somatosensory lesion [1]. Beyond widespread pain, patients report fatigue, unrefreshing sleep and cognitive difficulty, and the condition carries substantial disability and healthcare cost. The current literature converges on a central nervous system sensitized to both nociceptive and non-nociceptive input, with hypersensitivity extending to painful and nonpainful stimuli alike [2].

Affective processes occupy an unusual position within this account. They are regarded as central to the mechanism, and interventions that address emotional processing directly are among the more effective options available. In a randomized trial of 230 patients, emotional awareness and expression therapy performed comparably to cognitive-behavioural therapy, the field’s standard symptom-management intervention, on most outcomes. It did better on overall fibromyalgia symptoms (d = 0.35), widespread pain (d = 0.37) and the proportion of patients achieving 50% pain reduction (22.5% vs. 8.3%) [3]. Acceptance-based approaches are also recommended [2,4]. An integrative model attributes the condition to an imbalance between an overactive threat system and a hypoactive soothing system that holds the salience network in continuous alert. The same model identifies interpersonal rejection, whether actual, anticipated or only perceived, among the social signals shown to amplify pain in these patients [5]. Yet the instruments routinely recommended for assessment quantify affect as a level of symptom burden; that is, how depressed or anxious the patient currently is, and not as hypersensitivity, meaning how readily the patient responds with disproportionate distress to stimuli that are not themselves aversive. The affective limb of the amplification hypothesis is therefore treated but not measured.

The construct of emotional allodynia was proposed to fill this gap. It was originally defined as a qualitatively altered negative emotional response to normally non-aversive stimuli, demonstrated psychophysically with non-painful thermal stimulation in depression [6]. We transposed the construct from thermal to interpersonal stimuli of neutral or low intensity and operationalized it in the 11-item Emotional Allodynia Questionnaire (AEQ), whose preliminary validation in fibromyalgia showed a unidimensional structure and high internal consistency [7]. In a subsequent analysis, the association between the AEQ and difficulties in emotion regulation remained significant after simultaneous adjustment for depression, trait anxiety, pain catastrophizing and central sensitization, although the adjusted associations were of modest magnitude [8]. Whether emotional allodynia occupies a distinct position within the wider clinical structure of fibromyalgia, or is one more correlated index of distress, has not been examined.

This question is poorly suited to bivariate correlation, because in this population essentially every psychological measure correlates with every other. Network analysis offers a more informative framing: variables are represented as nodes, and each edge is the association between two variables conditional on all the others, so that indirect associations arising from shared neighbours are removed [9]. In practice, an edge between two questionnaires means that the two remain associated even when every other questionnaire in the model is held constant. Where no edge is drawn, no association survived that adjustment at this sample size. Within such a model, it becomes possible to ask not only how connected a variable is, but whether its connections cross the boundaries between clinical domains, which is quantified by bridge centrality [10].

Network analysis has been applied to fibromyalgia repeatedly, to psychophysical, sensory and physical-function variables [11,12], to executive function [13], and to depression together with cognitive performance [14,15,16]. The largest of them modelled 30 symptom nodes in 3044 patients and found depressed mood, anxiety, and fatigue to have the highest bridge centrality, while pain intensity ranked 18th [16]; a symptom-level network of 219 patients likewise found negative affect, but not pain, to be central [15]. That affective variables are the ones that connect the clinical domains is therefore a repeated finding. Which affective processes do so is less clear, because the affective domain has been represented by measures of symptom severity. The battery of the 3044-patient study comprised the Fibromyalgia Impact Questionnaire (FIQ), the SF-12 health survey and the PHQ-9, PHQ-15 and PHQ-stress modules of the Patient Health Questionnaire (PHQ). It also included a functional disability scale, a self-efficacy scale, the Center for Epidemiologic Studies Depression Scale (CES-D) and a pain-coping questionnaire [16]. In a twelve-node network of 50 patients, affect entered as depression, anxiety and perceived stress [17]. In the 219-patient network it entered as the PHQ-9 and the symptom subscale of the FIQ-R. None included a measure of emotion regulation, and none included one of affective hypersensitivity.

The present study estimates a regularized partial correlation network in a single-centre sample of patients with fibromyalgia. The battery separates depression, state and trait anxiety, pain catastrophizing, central sensitization and six facets of emotion dysregulation, and adds a measure of affective hypersensitivity. The aim is to identify which of these affective processes occupy the connecting positions of the network. We asked three questions. First, what position does emotional allodynia occupy in the network? Second, does its association with depression survive conditioning on the remaining variables? Third, is emotional allodynia distinguishable, in network terms, from pain catastrophizing, the construct to which it is conceptually closest?

## 2. Materials and Methods

### 2.1. Study Design and Participants

This is a cross-sectional secondary analysis of data collected within the PEARL study, an ongoing psychometric validation protocol for the Emotional Allodynia Questionnaire (AEQ) conducted at the tertiary Pain Clinic of Policlinico Hospital, University of Bari Aldo Moro, Bari, Italy. Consecutive adult outpatients attending the clinic between September 2025 and July 2026 were invited to participate; no patient was approached before ethical approval was granted on 8 September 2025. Eligibility required a diagnosis of fibromyalgia established by the treating physician according to the 2016 revision of the American College of Rheumatology criteria [18] and the ability to complete self-report questionnaires in Italian. Patients with cognitive impairment or with any current or past psychiatric diagnosis other than major depressive disorder and panic disorder were not enrolled. Psychotic disorders and bipolar disorder, among others, were excluded because they alter social cognition and emotional processing and would have confounded the measurement of affective responses to interpersonal cues. Major depressive disorder and panic disorder were not exclusion criteria, as they are highly prevalent in this population and excluding them would have distorted the clinical profile under study. All 149 participants were complete on all twelve variables, so no case was lost to missing data and the network was estimated on the full sample. No sample size was calculated for this analysis, which used every participant enrolled by the data cut-off; the adequacy of the sample for the estimated network was assessed through the stability analyses described in Section 2.6.

All participants provided written informed consent.

### 2.2. Relationship to Previous Reports from the Same Cohort

The present analysis draws on the same single-centre cohort as two previously published reports from the PEARL protocol. The first is the preliminary psychometric validation of the AEQ (*n* = 107) [7]; the second is an analysis of the association between emotional allodynia and specific facets of emotion dysregulation (*n* = 136) [8]. Because recruitment is continuous, the three samples represent successive snapshots of one cohort rather than independent samples, and the participants of the earlier reports are wholly contained within the present sample. Neither previous report estimated a network model. Both reported zero-order associations of the AEQ that reappear here, re-estimated in the larger sample, and the second also reported its partial correlations with the DERS facets (Supplementary Table S19). The present analysis adds the joint conditioning of all twelve variables, and with it the conditional association between the AEQ and the BDI-II, the distribution of connectivity across clinical domains and the comparison with pain catastrophizing. The three analyses should therefore not be counted as independent replications.

### 2.3. Measures

Twelve variables entered the network. Emotional allodynia was measured with the AEQ, an 11-item self-report instrument scored 0–44, in which higher scores indicate greater affective hypersensitivity to interpersonal stimuli that are neutral or of low intensity [7]. Emotional allodynia denotes disproportionate distress in response to interpersonal cues that are neutral or of low intensity; that is, a lowered threshold of emotional activation. It differs from pain catastrophizing, a set of cognitions about pain, and from emotion dysregulation, which concerns the management of an emotional response once it has arisen rather than the ease with which it arises. Pain catastrophizing was measured with the 13-item Pain Catastrophizing Scale (PCS) [19], and central sensitization with Part A of the Central Sensitization Inventory (CSI) [20]. Depressive symptom severity was measured with the Beck Depression Inventory-II (BDI-II) [21], and state and trait anxiety with the two forms of the State–Trait Anxiety Inventory, STAI-Y1 and STAI-Y2 [22]. Emotion dysregulation was measured with the 33-item Difficulties in Emotion Regulation Scale (DERS) [23]. Its six subscales (Non-Acceptance, Goals, Strategies, Impulse, Clarity and Awareness) were entered as separate nodes rather than as a total score, in order to resolve which facets of emotion regulation the AEQ connects to; this facet-level representation was planned before the network was estimated. The Italian adaptation was used [24], with 6, 5, 8, 6, 5 and 3 items, respectively, for Non-Acceptance, Goals, Strategies, Impulse, Clarity and Awareness. Higher scores indicate greater difficulty on all instruments. Internal consistency was estimated for each node from item-level responses, as Cronbach’s α and McDonald’s ω.

### 2.4. Network Estimation

A Gaussian graphical model was estimated, in which each edge represents the partial correlation between two nodes conditional on all remaining nodes [9]. Because several variables departed from normality, associations were computed as Spearman rank correlations, which are robust to skewed and ordinal-like score distributions. This is an alternative to the non-paranormal transformation used elsewhere [16], and was preferred because it depends only on the ranks of the scores; the two gave closely similar networks (Section 3.7). Because the partial correlations are computed from ranks, a zero edge means that no association between ranks survived conditioning on the other variables, and it is not read as conditional independence in the sense that applies to a multivariate normal distribution. The same choice, for the same reason, was made in a recent network analysis of fibromyalgia and depression [15]. The model was regularized with the graphical LASSO, with the penalty parameter selected by the Extended Bayesian Information Criterion (EBICglasso) with hyperparameter γ = 0.5, the value recommended for favouring specificity [9]. Regularization shrinks small partial correlations to exactly zero, yielding a sparse and more interpretable network. With twelve nodes there are 66 candidate edges for 149 observations, so the estimates are subject to appreciable shrinkage and an edge of zero is the outcome of a selection decision rather than an unbiased estimate of no association.

The network was visualized with the Fruchterman–Reingold force-directed layout, in which node placement carries no quantitative meaning. Node predictability, defined as the proportion of variance of each node explained by all of its neighbours, was computed with mixed graphical models (neighbourhood selection by extended Bayesian information criterion; lambdaSel = “EBIC”, lambdaGam = 0.5). All twelve variables were specified as Gaussian, and the metric reported is R^2^. This model estimates its own nodewise neighbourhoods and is therefore not identical to the displayed network; predictability is reported as a descriptive companion to it rather than as a parameter of it, and the predictors it retained were compared with the edges of the displayed network [25].

### 2.5. Centrality and Bridge Centrality

Strength centrality was computed as the sum of the absolute weights of the edges incident to each node. Betweenness and closeness were computed but are not reported, being markedly less stable in cross-sectional networks [9] and of questionable suitability for networks of this kind; for this reason, they have been excluded altogether in this recent network analysis [16].

Bridge strength, defined as the sum of the absolute weights of the edges connecting a node to nodes outside its own community [10], was computed with respect to four communities assigned a priori on substantive grounds; that is, by domain of measurement rather than from the data (the partition was not registered in advance; see Section 4.4). This follows the convention in bridge-centrality research where communities are defined by clinical meaning rather than by covariance, since the question of interest concerns domains of measurement. Where nodes are individual symptoms shared between two diagnoses, theoretical assignment is not possible and data-driven detection is the appropriate choice [16]; our nodes are total scores of distinct validated instruments, for which assignment to a domain is unambiguous. The four communities were emotional allodynia (AEQ), pain (PCS, CSI), depression and anxiety (BDI-II, STAI-Y1, STAI-Y2), and emotion regulation (the six DERS subscales). Two consequences of this choice were addressed directly. First, because the AEQ is the focal construct, it forms a community of one, so all of its edges are between-community and its bridge strength is by construction equal to its total strength. Its value is therefore not comparable with that of nodes that lose their within-community edges. We therefore report bridge strength only descriptively. Interpretation rests instead on how each node’s connectivity is distributed across the domains, that is, the percentage of the total weight of its edges that goes to each domain (Supplementary Table S5). Unlike bridge strength, this percentage is not affected by the AEQ forming a community of one. Second, as a sensitivity analysis, we compared the a priori partition with data-driven community detection (walktrap, spinglass and Louvain algorithms on the absolute-weight graph).

### 2.6. Accuracy, Stability and Redundancy Analyses

Accuracy of edge weights was assessed by non-parametric bootstrapping with 5000 replications. The stability of centrality estimates was assessed by case-dropping subset bootstrapping and summarized by the correlation-stability (CS) coefficient, computed at a reference correlation of 0.70 and reported separately for strength and for bridge strength. Values above 0.25 are considered the minimum acceptable values and values above 0.50 are preferable [9]. Because bridge strength is not covered by the default bootnet case-dropping routine, its CS coefficient was computed separately. Its sampling variability was quantified with an additional non-parametric bootstrap of 5000 replications, in which the network was re-estimated in each resample and bridge strength recomputed, yielding percentile intervals. Because bootstrapped sampling distributions of centrality indices are biased, these intervals describe sampling variability and are not true 95% confidence intervals [26].

Topological redundancy between nodes was assessed with the goldbricker procedure, which compares the correlation profiles of node pairs and flags pairs whose profiles are insufficiently distinct [27] with a threshold of 0.25 and a minimum correlation of 0.50. Where redundancy was flagged for a pair of substantive interest, the two nodes were compared in two ways. First, the difference between their dependent correlations was tested. Second, for each remaining node two partial correlations were computed and compared: that of the AEQ with the node, adjusted for the PCS, and that of the PCS with the same node, adjusted for the AEQ. The difference between them was assessed with 5000 bootstrap replications. Because an edge shrunk to zero by the LASSO penalty is not evidence of conditional independence, absent edges of substantive interest were examined post hoc by means that do not depend on LASSO selection. Three approaches were used. The first recomputed the unpenalized partial Spearman correlation of the pair across a nested sequence of adjustment sets, with percentile intervals from a non-parametric bootstrap of 5000 replications. The second compared rank-based linear models of the AEQ with and without the node in question, by BIC, by the increase in R^2^ and by ten-fold cross-validation repeated 100 times. The third assessed equivalence to zero with two one-sided tests across a range of margins; no minimum relevant effect size had been specified in advance. The unpenalized partial correlation between the AEQ and the BDI-II was also recomputed after adjustment for, and within strata of, documented depressive disorder and current antidepressant use.

### 2.7. Sensitivity Analyses and Software

Sensitivity analyses were performed at two levels. Three concerned the estimation itself. The first re-estimated the network with thresholding enabled, which retains only edges that survive a more conservative specificity criterion. The second used γ = 0.25, in order to match the specification of the previously published network analysis of fibromyalgia with which our findings are compared [17]. The third applied a nonparanormal transformation. Two concerned the representation of the instruments, since the share of connectivity a domain receives depends on how many nodes it contains. The network was re-estimated with the PCS entered as its three subscales, which enlarges the pain domain. It was then re-estimated with the six DERS facets replaced by the DERS total score, which reduces the emotion-regulation domain to a single node. The domain shares of the main network were also normalized by the number of nodes each domain makes available. All analyses reported in this paper are exploratory. No analysis plan was registered in advance, no correction for multiplicity was applied, and bootstrap intervals are reported to describe the precision of the estimates rather than to support confirmatory inference.

All analyses were performed in R 4.5.1 with qgraph 1.9.8, bootnet 1.8, networktools 1.6.0, mgm 1.2.15, psych 2.6.3, ppcor 1.1 and huge 2.0.1 [28]. Fixed random seeds were used throughout, set separately for the network estimation and difference tests and for the additional bridge-strength bootstrap, and recorded in the analysis scripts. Analysis scripts are available as described in the Data Availability Statement. Reporting follows current recommendations for psychological network analyses in cross-sectional data [29].

### 2.8. Ethics

The study was conducted in accordance with the ethical principles of the Declaration of Helsinki and with the International Association for the Study of Pain (IASP) guidelines for pain research in humans. Ethical approval was obtained from the Local Ethics Committee of the IRCCS Oncological Institute “Gabriella Serio”, IRCCS “Giovanni Paolo II”, Bari, Italy (approval number 2451/CEL, approved on 8 September 2025). All participants provided written informed consent prior to enrolment. Data were pseudonymized at collection and no identifying information was accessible to the analysts. The PEARL protocol is registered at ClinicalTrials.gov (NCT07677631); the present report concerns the single-centre phase, whose data collection preceded that registration.

## 3. Results

### 3.1. Sample Characteristics

The analytic sample comprised 149 outpatients with fibromyalgia, 91.9% of them women, with a mean age of 57.5 years (range 33–85). Their demographic, clinical and interpersonal characteristics are reported in Table 1, and descriptive statistics for the twelve network nodes in Table 2. Complete data on all twelve variables were available for all 149 participants, so no imputation was required. Skewness ranged from −0.29 to 1.02, DERS Impulse being the most positively skewed node. Floor effects were present in the emotion-regulation nodes, 25.5% of participants scoring the minimum on DERS Awareness and between 2.0% and 14.1% doing so on the other five facets; no node showed a ceiling effect. Internal consistency was acceptable to excellent for eleven nodes and fell below the conventional threshold for DERS Clarity (α = 0.661; Supplementary Table S1).

### 3.2. Network Structure, Predictability and Centrality

The regularized partial correlation network is displayed in Figure 1. Of the 66 possible edges, 43 (65.2%) were estimated as non-zero, with 39 of them exceeding an absolute weight of 0.05 and 27 exceeding 0.10. Of these 43 edges, 40 were positive and 3 were negative, the negative edges being those of DERS Awareness with the PCS, with DERS Non-Acceptance and with DERS Goals. Edges appeared more concentrated within the a priori depression-and-anxiety and emotion-regulation domains than between them. The two pain instruments were placed apart, with the CSI lying closer to the depression and anxiety nodes and the PCS closer to the emotion-regulation ones. Because that is a visual impression of a force-directed layout, the quantitative counterparts are the domain-connectivity proportions of Supplementary Table S5 and the data-driven community detection reported in Section 3.3.

The strongest edges in the network were observed within clusters, most notably between the two anxiety scales and among the DERS facets, consistent with conceptual and measurement overlap between these instruments. The full weighted adjacency matrix is provided as Supplementary Table S2.

Node predictability, computed with the mixed graphical model described in Section 2.4, averaged R^2^ = 0.445 across the twelve nodes (range 0.058 to 0.693). Predictability was highest for STAI-Y2 (R^2^ = 0.693) and STAI-Y1 (R^2^ = 0.668), and lowest for DERS Awareness (R^2^ = 0.058) and DERS Non-Acceptance (R^2^ = 0.197). Predictability for the AEQ was R^2^ = 0.402. Because the two STAI forms correlate at ρ = 0.869, each is largely predictable from the other; with state anxiety removed from the model, the predictability of trait anxiety fell to R^2^ = 0.469, while that of the AEQ was unchanged (Supplementary Table S15). Every predictor retained by the nodewise models was also an edge of the displayed network (Supplementary Table S21).

Standardized centrality indices are reported in Supplementary Table S3. The highest estimated strength values were those of STAI-Y2 (z = 1.50), DERS Strategies (z = 1.32) and BDI-II (z = 0.88). The bootstrap difference test did not distinguish them from one another (STAI-Y2 versus DERS Strategies, difference 0.039, 95% percentile interval −0.224 to 0.284; Figure S1c). Their relative ordering is therefore not interpreted. The AEQ was among the less connected nodes (z = −0.44): its strength was lower than that of STAI-Y2 (percentile interval of the difference −0.647 to −0.090) and of DERS Strategies (−0.611 to −0.077). It fell in the lower half of the ranking in 81.3% of the 5000 replications. Because the twelve instruments are strongly intercorrelated, we also examined whether their covariance is reducible to a single dimension. A single common factor accounted for 48.7% of the variance, but parallel analysis indicated three factors rather than one and the single-factor solution did not fit adequately (Tucker–Lewis index 0.839; seven of the 66 off-diagonal residuals above 0.10). Loading on that factor was nonetheless correlated with strength centrality at r = 0.745 across the twelve nodes (Supplementary Table S13).

### 3.3. Distribution of Connectivity Across Clinical Domains

Bridge strength with respect to the four a priori communities is reported in Supplementary Table S4, with bootstrap percentile intervals (2.5th–97.5th) from 5000 non-parametric replications. These values are reported descriptively and are not ranked against one another: because the AEQ forms a community of one, its bridge strength is by construction identical to its total strength (0.815), whereas every other node forfeits its within-community edges. The comparison we interpret is therefore the one presented in Figure 2 and Supplementary Table S5, which is unaffected by this asymmetry.

Figure 2 gives the proportion of each node’s total connectivity directed at each domain, with the underlying values and their bootstrap percentile intervals in Supplementary Table S5. The AEQ’s connectivity was directed at emotion regulation (59.8%, 95% percentile interval 35.5 to 82.6) and at the pain domain (30.2%, 7.3 to 51.5), and only marginally at depression and anxiety (10.0%, 0.0 to 28.4). These three domains contain six, two and three of the eleven remaining nodes, respectively. The shares reported here are not corrected for these different sizes; shares normalized by the number of nodes are reported in Section 3.7. The pain domain comprises the two pain questionnaires, pain intensity itself not being a node (Section 3.4). The relative ordering of the first two domains depends on how finely each of them is represented and is examined in Section 3.7; the contrast between both of them and depression and anxiety does not depend on this. Two between-node comparisons follow. Of all nodes outside the pain domain, the AEQ directed the largest share to it, and it retained the largest such share in 48.1% of the 5000 replications. Of all nodes outside the emotion-regulation domain, it likewise directed the largest share to that one, 59.8% against 45.1% for pain catastrophizing and 40.7% for the BDI-II, and it retained that share in 77.0% of the replications. Its share directed at depression and anxiety was smaller than that of pain catastrophizing (24.6%) and of central sensitization (48.2%).

The CSI, an instrument of the pain domain, correlated with depression (ρ = 0.570) and with the two anxiety scales (0.565 and 0.548) as strongly as with the other pain instrument (ρ = 0.487; Supplementary Table S6). In the network its edge with the BDI-II (0.146) was numerically larger than that with pain catastrophizing (0.101), although the bootstrap did not distinguish the two, and its largest edge was with DERS Goals (0.163). Data-driven community detection, with the three algorithms of Section 2.5 returning identical partitions, recovered two communities rather than four (adjusted Rand index 0.491 against the a priori partition). It grouped the CSI with depression and both anxiety scales and placed the AEQ, the PCS and the six DERS facets together. Under that partition, the bridge strength of the AEQ falls from 0.815 to 0.138, because the edges that carry most of its connectivity, those with the PCS and the DERS facets, no longer cross a community boundary.

### 3.4. Direct Associations of the AEQ

Table 3 contrasts the zero-order Spearman correlations of the AEQ with its regularized partial correlations (network edges). At the zero-order level, the AEQ correlated significantly with ten of the eleven remaining variables (ρ = 0.44–0.60, all *p* < 0.001), the sole exception being DERS Awareness (ρ = 0.049, *p* = 0.556). The full zero-order matrix for all twelve nodes is given in Supplementary Table S6. Current pain intensity, recorded for all participants (Table 1) but not entered as a network node, was uncorrelated with the AEQ (ρ = −0.012, *p* = 0.887).

Once all other variables were conditioned upon, seven edges remained non-zero, the strongest with the PCS (0.190) and DERS Impulse (0.184). Four fell to exactly zero: DERS Awareness, DERS Goals, STAI-Y1 and, most notably, the BDI-II, whose zero-order correlation with the AEQ had been ρ = 0.502 (*p* < 0.001).

Across 5000 bootstrap replications, the seven non-zero edges were estimated as non-zero in 74.0–99.6% of samples and the four zero edges in only 26.0–35.3%, with no overlap between the two; full inclusion proportions are given in Supplementary Table S7. Unpenalized partial Spearman correlations, controlling simultaneously for the other ten variables, gave the same picture: only the edges with the PCS (ρ = 0.191, *p* = 0.025) and DERS Impulse (ρ = 0.174, *p* = 0.041) reached nominal significance, uncorrected for the eleven comparisons. The estimates for the BDI-II (ρ = −0.033, *p* = 0.703) and state anxiety (ρ = −0.038, *p* = 0.661) were close to zero. Because the null edge with the BDI-II carries much of the interpretation that follows, it was examined further by means that do not depend on LASSO selection (Supplementary Tables S17 and S18). Across a nested sequence of adjustment sets, the association fell in two steps: adjusting for pain catastrophizing alone halved it (ρ = 0.251), and further adjusting for the six DERS facets removed almost all of what remained (ρ = 0.044). The two anxiety scales and the CSI together changed it by less than 0.08 (ρ = −0.015 and −0.033). The bootstrap percentile interval of the full-model partial correlation was −0.229 to 0.162, and the upper one-sided 95th percentile for a positive residual association was 0.130. Adding the BDI-II to a rank-based model of the AEQ raised BIC by 4.845, an approximate evidence ratio of 11.3 to 1 in favour of the model without it, raised R^2^ by 0.00054, and improved cross-validated prediction in none of 100 repeated ten-fold cross-validations. Equivalence to zero was not reached at a margin of |ρ| < 0.15 (*p* = 0.084) and was reached only at |ρ| < 0.20 (*p* = 0.024). The estimate changed little after adjustment for documented depressive disorder and current antidepressant use (ρ = −0.039), and the estimates within strata, although imprecise, gave no indication of a different association (Supplementary Table S20).

### 3.5. Accuracy, Stability and Redundancy

Non-parametric bootstrapping (5000 replications) indicated moderate accuracy of edge-weight estimates; bootstrapped confidence intervals were relatively wide, and the edge-difference test showed that only the strongest edges could be reliably distinguished from one another (Supplementary Figure S1a–c).

Case-dropping subset bootstrapping yielded correlation-stability (CS) coefficients of 0.517 for both strength centrality and bridge strength. These exceed the minimum of 0.25 and reach the 0.50 value regarded as preferable, but indicate moderate rather than high stability. Relative centrality orderings are therefore interpreted cautiously.

Twelve node pairs were flagged as showing insufficiently distinct correlation profiles, including AEQ–PCS and AEQ–DERS Impulse. The pairs flagged also included STAI-Y2 with BDI-II, STAI-Y1 with BDI-II and DERS Goals with DERS Non-Acceptance (proportion of significantly different correlations 0.1–0.2; Supplementary Table S8).

### 3.6. Discriminating the AEQ from Pain Catastrophizing

The AEQ and the PCS correlated at ρ = 0.570 (ρ^2^ = 0.325). Comparison of their correlation profiles across the remaining ten nodes yielded no statistically significant difference (all |Δρ| ≤ 0.087, all *p* ≥ 0.154). The differences were nonetheless consistent in direction. Relative to the PCS, the AEQ correlated less strongly with depression (0.502 vs. 0.589) and state anxiety (0.506 vs. 0.590) and more strongly with difficulties in emotional clarity (0.444 vs. 0.358).

The two nodes nonetheless differed in which edges the network retained. Their connectivity profiles pointed in different directions with respect to the depression and anxiety domain (10.0% for the AEQ against 24.6% for the PCS; Supplementary Table S5). At the level of individual edges (Table 4) the PCS was connected to the BDI-II (0.114) and to state anxiety (0.121), whereas both of these edges were exactly zero for the AEQ. Conversely the AEQ was connected to trait anxiety (0.082) and to DERS Clarity (0.078), the latter the least reliable node in the network (α = 0.661; Supplementary Table S1), both of which were zero for the PCS.

This dissociation was examined further with partial correlations in which each of the two measures was adjusted for the other (Supplementary Table S9). The AEQ retained a significant unique association with difficulties in emotional clarity, which the PCS did not, whereas the PCS retained a substantially stronger unique association with depression and state anxiety. Both measures retained significant unique associations with DERS Impulse, Strategies, Non-Acceptance and Goals, and neither was associated with DERS Awareness. These contrasts were descriptive: the difference between the two measures was not statistically established.

### 3.7. Sensitivity Analyses

The network was re-estimated with threshold = TRUE. This reduced the number of non-zero edges from 43 to 15. Across all 66 edges, the two solutions correlated r = 0.834, while the fifteen retained weights correlated r = 0.986 with their main-analysis values. They were not identical to them, changing by between −0.017 and +0.066 (Supplementary Table S10). The thresholded network was a strict subset of the main one, so this analysis can only remove edges and provides no independent information about edges already estimated at zero. Five of the seven AEQ edges were removed, among them those with trait anxiety and with DERS Clarity on which the comparison with pain catastrophizing in Section 3.6 rests. The two that survived were those with the PCS (0.185) and with DERS Impulse (0.184), one from the pain cluster and one from the emotion-regulation cluster.

The network was also re-estimated with the EBIC hyperparameter set to γ = 0.25. The resulting network was identical to the main solution (r = 1.000; 43 non-zero edges), and all reported findings were unchanged (Supplementary Table S11). Re-estimation after a nonparanormal transformation gave a highly similar network (r = 0.986; Supplementary Table S12). Finally, because the domain proportions depend on how many nodes each domain contains, and the DERS was entered at facet level while the other instruments were entered as total scores, the network was re-estimated with the PCS entered as its three subscales, which enlarges the pain domain from two nodes to four. The share the AEQ directed at that domain moved from 30.2% to 33.6% and the share directed at depression and anxiety from 10.0% to 9.6%, and its edge with the BDI-II remained exactly zero. The opposite manipulation was performed as well, with the six DERS facets being replaced by the DERS total score, which reduces the emotion-regulation domain from six nodes to one. Under that representation, the ordering of the first two domains reversed: the share the AEQ directed at the pain domain rose to 45.0% and the share directed at emotion regulation fell to 41.1%. The share directed at depression and anxiety remained the smallest at 13.8%, and the edge with the BDI-II again remained exactly zero. Normalizing the shares of the main network by the number of nodes each domain makes available gives the same picture (pain 53.2%, emotion regulation 35.1%, depression and anxiety 11.7%). The four representations are set out side by side in Supplementary Table S16. Of the three catastrophizing subscales, the AEQ was connected to Magnification (0.171) and, weakly, to Helplessness (0.062), while its edge with Rumination was zero (Supplementary Table S14). These three edges were re-estimated in 5000 bootstrap replications: the Magnification edge was non-zero in 96.7% of them, the Helplessness edge in 74.4% and the Rumination edge in 42.1%. The percentile interval of the difference between the Magnification and Rumination edges was −0.047 to +0.295, which includes zero (Supplementary Table S14). The ordering of the three subscales is not established at this sample size; what the subscale analysis does establish is that the zero edge with the BDI-II survives this change in granularity as well.

## 4. Discussion

In this exploratory analysis of twelve clinical dimensions measured in 149 patients with fibromyalgia, a cohort that includes the participants of two previous reports (Section 2.2), emotional allodynia stood apart from the other measures. On its own it correlated substantially with depression. In the network, where each association is adjusted for the other ten measures, that association fell to zero, and it stayed at or close to zero in every check we ran, including those without regularization (Section 3.4 and Section 3.7). Emotional allodynia had few strong connections overall (strength centrality), and those it kept ran to emotion regulation and to pain catastrophizing. Three observations follow in decreasing order of how firmly these data support them.

### 4.1. Emotional Allodynia and Depression: An Association That Does Not Survive Conditioning

The disappearance of the AEQ–depression association under conditioning is the most readily interpretable of our findings. A zero-order correlation of 0.502 would ordinarily be read as evidence that a new affective questionnaire is measuring depressed mood. The network shows instead that nothing distinguishable from zero remains once the other measured variables are held constant, although the model does not identify the pathways involved. Two patients with the same depression score can differ widely in emotional allodynia. Among patients who were similar on the other questionnaires, the depression score gave no further information about emotional allodynia. This is consistent with the partial-correlation findings we reported previously in a subset of this cohort [8] and with the discriminant validity evidence from the original validation [7].

The analyses reported in Section 3.4 go further than the unselected edge. A model that omits the contribution of depressive symptom severity is favoured over one that retains it by an approximate BIC evidence ratio of about eleven to one; that is, there is about eleven times more support for leaving depression out of the model than for keeping it in. Adding it never improved prediction in patients left out of the model. This is relative evidence in favour of omitting the association, not merely a failure to select an edge. The claim has a limit on each side. A regularized edge of zero means that the association is not distinguishable from zero at this sample size under this penalty, not that conditional independence has been demonstrated, and a larger sample could recover a small edge. Equivalence was not reached at margins of 0.15 or smaller, and what these data exclude is a positive residual association larger than about 0.13, roughly a quarter of the raw correlation of 0.502. In this battery, therefore, emotional allodynia showed little residual association with depressive symptom severity once the other measured variables were held constant. Whether that absence carries clinical information, for example, on prognosis or on the response to treatment, was not tested.

### 4.2. Where Emotional Allodynia Sits in the Network

The Central Sensitization Inventory, the instrument intended here to capture sensory amplification, correlated no more strongly with the other pain instrument than with the depression and anxiety measures. Data-driven community detection grouped it with those measures rather than with pain. The CSI records symptoms that co-occur with central sensitization rather than a response to a specified stimulus, so a substantial affective loading is unsurprising: in a meta-analysis of 66 studies, it correlated strongly with depression, anxiety and pain catastrophizing [30]. This may signal that the affective dimension of fibromyalgia is not cleanly separated from its pain measures in the batteries currently in use, which is an argument for measuring affective hypersensitivity directly.

Asked of whole networks rather than of a single instrument, the same question has a well-supported answer: affective variables occupy the connecting positions of the fibromyalgia network, in samples ranging from 50 to 3044 patients [15,16,17]. The present results are consistent with it. The three nodes with the highest strength were affective: trait anxiety, emotion-regulation strategies and depressive symptom severity. We do not interpret the ordering among them (Section 3.2). What those studies could not address is which affective processes occupy those positions, because in those studies affect was represented by symptom-severity measures (Section 1).

When that region is instead resolved into six facets of emotion dysregulation together with a measure of hypersensitivity to neutral and low-intensity cues, depressive symptom severity no longer exhausts the affective contribution. The apparent centrality of depression in earlier work may partly reflect which instruments were in the model. This fits earlier evidence that the psychological profile of fibromyalgia is multidimensional, with patient subgroups that combine mood, pain catastrophizing and tenderness in different ways [31], and with catastrophizing, anxiety and depressive symptoms showing different candidate-gene associations in a cross-sectional study [32].

The connectivity of emotional allodynia ran to how patients handle their emotions (59.8% of it) and how they appraise pain (30.2%), and showed little relation to how depressed or anxious they were (10.0%). The domain to which that connectivity is attributed depends on how the domains are drawn, and this is the weakest link in the analysis. Communities were defined a priori by domain of measurement, as is conventional in bridge-centrality research. Under that partition the AEQ forms a community of one, so all of its connectivity crosses a boundary by construction and its bridge strength of 0.815 is merely its total strength restated. That value is uninformative and we do not rank nodes on it.

The data-driven partition is the more informative comparison: it recovered two communities rather than four (adjusted Rand index 0.491), placing the AEQ alongside pain catastrophizing and the six DERS facets, and the bridge strength of the AEQ fell accordingly to 0.138. The edges are unchanged, so this is a reclassification of the same network rather than a different result. It is the reading we prefer: emotional allodynia belongs to a block of pain-related and emotion-regulatory dimensions, which is what a general amplification of central reactivity in fibromyalgia would predict. Its zero edge with the BDI-II does not depend on the partition, and the small share of connectivity it directs at the three depression and anxiety measures persisted when the other domains were represented more or less finely (Section 3.7).

Among the six DERS facets, the strongest direct connection of the AEQ was with Impulse, difficulty maintaining behavioural control when distressed, followed by Strategies. Awareness showed a negligible zero-order correlation (ρ = 0.049) and a zero edge. Patients high in emotional allodynia are therefore those who find it hard to control their behaviour when they are distressed, rather than those who do not notice their emotions. The same ordering was found using partial correlations in a subset of this cohort [8]. Because 136 of the present 149 participants are those of that report, this is a dependent re-analysis of largely the same material under a different conditional model and not a replication. Whether the dissociation identifies a therapeutic target was not tested here.

Node predictability, the share of a score that the other questionnaires can account for, points the same way. The remaining eleven variables jointly accounted for 40.2% of the variance in AEQ scores (the identical 59.8% reported earlier is the share of connectivity, a different quantity), against 66.8% and 69.3% for the two anxiety scales. That contrast is smaller than it appears, because the two STAI forms correlate at ρ = 0.869: with only one anxiety scale in the model, the predictability of trait anxiety falls to 0.469, while that of the AEQ is unchanged (Supplementary Table S15). In this sample, therefore, most of the variance in AEQ scores was not accounted for by the other instruments in the battery. Its comparatively low strength centrality is consistent with this, not against it: loading on the single common factor of Section 3.2 tracks strength closely, so a node low on strength is among the less saturated indicators of general distress. That is the expected direction for such an instrument, though not in itself evidence that it succeeds. That emotional allodynia was uncorrelated with current pain intensity argues against its scores simply reflecting pain severity, although the ceiling of that single item limits the comparison.

### 4.3. Emotional Allodynia and Pain Catastrophizing

The AEQ and the PCS correlated at ρ = 0.570, and a formal redundancy procedure (goldbricker) flagged them as insufficiently distinct.

The procedure also flagged the BDI-II with both forms of the STAI and two DERS facets with each other, pairings that would imply a single construct underlying separately validated inventories. This same procedure was developed for item-level networks, where near-identical items are the target, and it compares dependent correlations, which are underpowered at this sample size. The flag is a caution against strong claims of distinctness rather than evidence of interchangeability. The two measures share less than a third of their rank variance (ρ^2^ = 0.325, an index of shared rank ordering rather than of variance explained), a ratio we have previously used against the same objection with respect to a DERS facet [8].

At the level of the whole scales, the two nodes retained edges with different neighbours, catastrophizing to depression and to state anxiety, and emotional allodynia to trait anxiety and to emotional clarity (Table 4). Adjusted for each other, only the AEQ retained a unique association with clarity, while the PCS retained one with depression roughly twice the size of the AEQ’s. These contrasts are descriptive: the edge-difference test did not distinguish the pairs. The AEQ directs 30.2% of its connectivity at the pain domain, and more than three quarters of that share is the single edge with pain catastrophizing (23.3% of its total connectivity).

Resolving catastrophizing into its components sharpens the comparison without settling it. Of the three PCS subscales, the edge with Magnification was the largest and the most consistently retained while the edge with Rumination was zero. That ordering is what an account in which an overactive threat system holds the salience network in continuous alert [5] would predict, since the Magnification items concern the anticipation of harm rather than preoccupation with pain already present. But a Rumination edge was still selected in 42.1% of replications and the interval of the difference between the two edges includes zero (Section 3.7), so the ordering is suggestive and not established.

These data do not adjudicate the separability of the two constructs. A total score cannot express whether the overlap is anticipatory or ruminative, and that comparison is a natural target for the multicentre phase of this protocol, where it can be adequately powered. These data establish neither that the two instruments are distinct nor that they are interchangeable.

### 4.4. Limitations

The design is cross-sectional, the edges are undirected, and nothing in this analysis licenses causal or temporal language: the position of a node describes a covariance structure at one point in time, not a mechanism. The correlation-stability coefficient was 0.517 for both strength and bridge strength, meeting but not comfortably exceeding the recommended value; we therefore interpret centrality alongside the spread of the bootstrap distributions and do not interpret small differences in ordering. The corresponding value in the 3044-patient network was 0.75 for bridge strength [16].

The zero edge between emotional allodynia and depression is conditional on the particular set of the other ten variables entered here: the argument we make in Section 4.2 about the earlier networks applies to ours. Results that depend on the community partition require the caution set out there. Adjusted for pain catastrophizing alone, that association is 0.251 (*p* = 0.002), so what the network shows is that no residual association was detected once catastrophizing, anxiety and emotion dysregulation were held constant, not that the two are unrelated. A further explanation cannot be separated in these data: 45.6% of the cohort carried a documented depressive disorder and 38.9% were taking an antidepressant. The BDI-II asks about the preceding two weeks, whereas the AEQ asks how often interpersonal situations are experienced in a given way, so a treated patient may report attenuated recent depressive symptoms alongside an unchanged relational style.

The sample was recruited at a single centre and was 91.9% female, which reflects the epidemiology of fibromyalgia but precludes analysis by sex and limits generalizability. Selection at a tertiary centre may restrict the range of clinical severity and thereby affect the associations estimated between nodes. Excluding psychiatric disorders other than major depressive disorder and panic disorder may also have narrowed the variance of emotion regulation, anxiety and emotional allodynia, and the associations estimated here may not extend to patients with those conditions.

The network model, the community partition and the decomposition of catastrophizing into its subscales were not pre-specified: the protocol registration followed data collection, and no analysis plan for these questions was lodged in advance. The sensitivity analyses show which results hold across specifications and which do not, but they cannot convert an exploratory analysis into a confirmatory one, and every result reported here should be read as hypothesis-generating. The sample overlaps with two previous reports from the same cohort (Section 2.2). Our battery lacks the measures of fatigue, sleep and functional impact present in the earlier fibromyalgia network [17], so the two studies cover overlapping but not identical clinical territory and neither is a replication of the other. Pain intensity was recorded but not entered as a node, being a single item with a marked ceiling rather than a multi-item score like the twelve nodes. Finally, the AEQ is a recently developed instrument whose validation remains preliminary. Its scores occupy a distinct conditional position in this network, but whether emotional allodynia is a clinical construct independent of the others can only be established in independent samples and against external criteria.

## 5. Conclusions

In a network model of an affective and pain-related questionnaire battery in tertiary-care fibromyalgia, emotional allodynia was sparsely connected overall. Its connectivity was nonetheless directed towards the pain domain and towards emotion regulation rather than towards depression and anxiety, and its zero-order association with depression did not survive conditioning on the remaining variables. Emotional allodynia and pain catastrophizing overlapped substantially, and these data do not adjudicate their separability; nor does this design, which includes no independent outcome, test whether measuring emotional allodynia predicts outcome or informs treatment choice. Both questions fall to the ongoing multicentre phase of the PEARL protocol, which is intended to supply the replication these preliminary findings require. What these data suggest is narrower, and conditional on the variables measured. In a battery that separates them, and that does not include fatigue, sleep or functional impact, the affective dimension of fibromyalgia is not exhausted by depression and anxiety severity, and emotional allodynia may carry information that depressive symptom severity does not fully represent. In clinical terms, in this sample, how depressed a patient is and how strongly that patient reacts to minor interpersonal cues appear to be two different pieces of information.

## Figures and Tables

**Figure 1:**
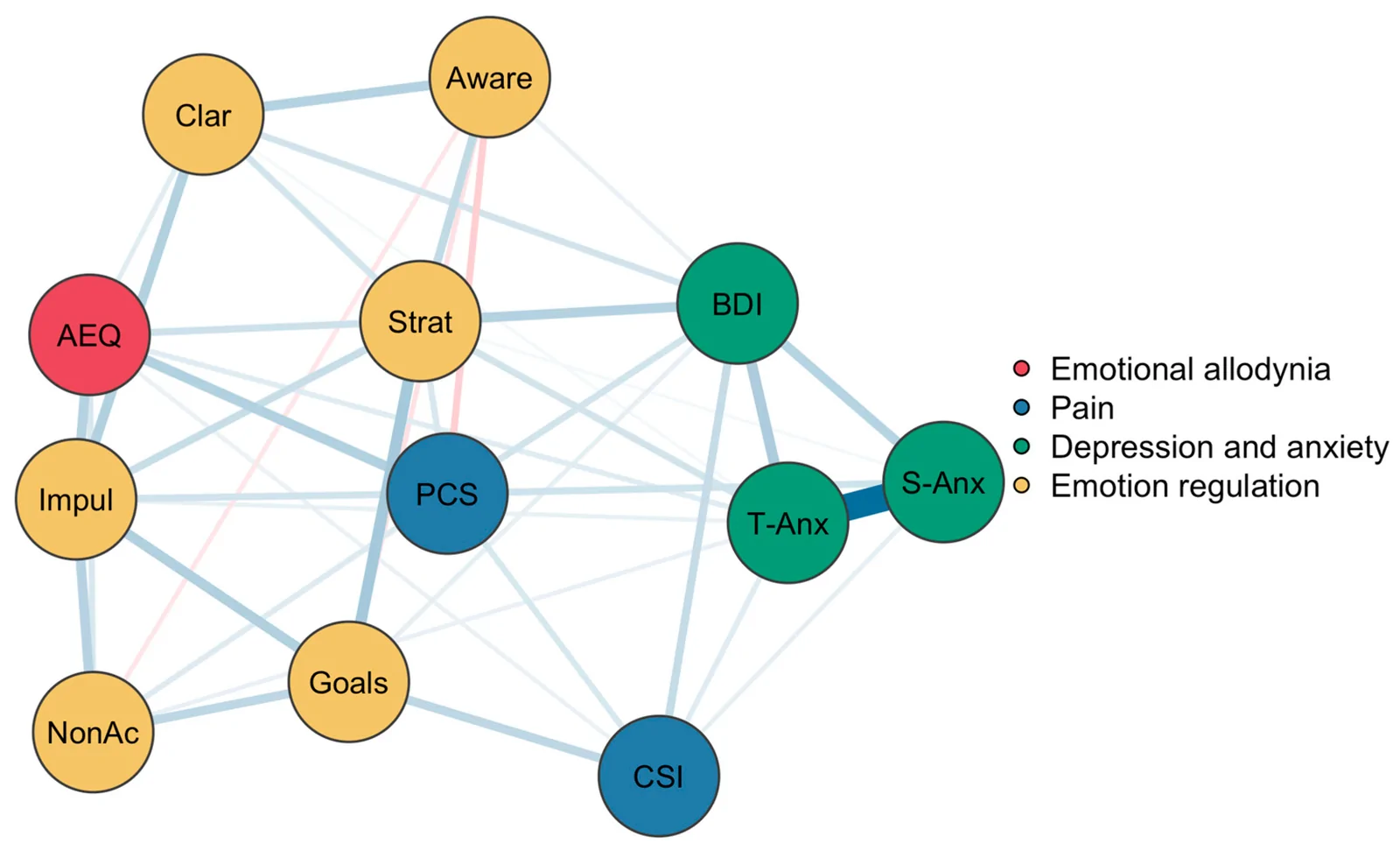
Regularized partial correlation network (EBICglasso, γ = 0.5, Spearman correlations) of emotional allodynia, pain, depression and anxiety, and emotion-regulation variables in 149 patients with fibromyalgia. How to read the figure: each node is one questionnaire score. Each edge is the partial correlation between the two nodes it joins, after conditioning on all ten remaining variables. An edge therefore represents an association that the other ten measured variables do not account for; associations with variables outside the model are not controlled. Edge thickness and colour saturation are proportional to the absolute weight of that partial correlation; blue edges are positive and red edges negative. Node colour denotes the a priori community to which the node was assigned for the computation of bridge centrality. The absence of an edge indicates an association shrunk to zero by the LASSO penalty, which is not evidence of conditional independence. Node placement follows a force-directed (spring) layout: more strongly connected nodes are drawn closer together, but distances and absolute positions carry no quantitative meaning and should not be interpreted. Of the 66 possible edges, 43 were non-zero; the strongest edge was 0.583 (state–trait anxiety) and three edges were negative. Abbreviations: AEQ—Emotional Allodynia Questionnaire; PCS—Pain Catastrophizing Scale; CSI—Central Sensitization Inventory; BDI—Beck Depression Inventory-II; S-Anx and T-Anx—STAI-Y1 (state) and STAI-Y2 (trait) anxiety; NonAc, Goals, Strat, Impul, Clar and Aware—the Non-Acceptance, Goals, Strategies, Impulse, Clarity and Awareness subscales of the DERS.

**Figure 2:**
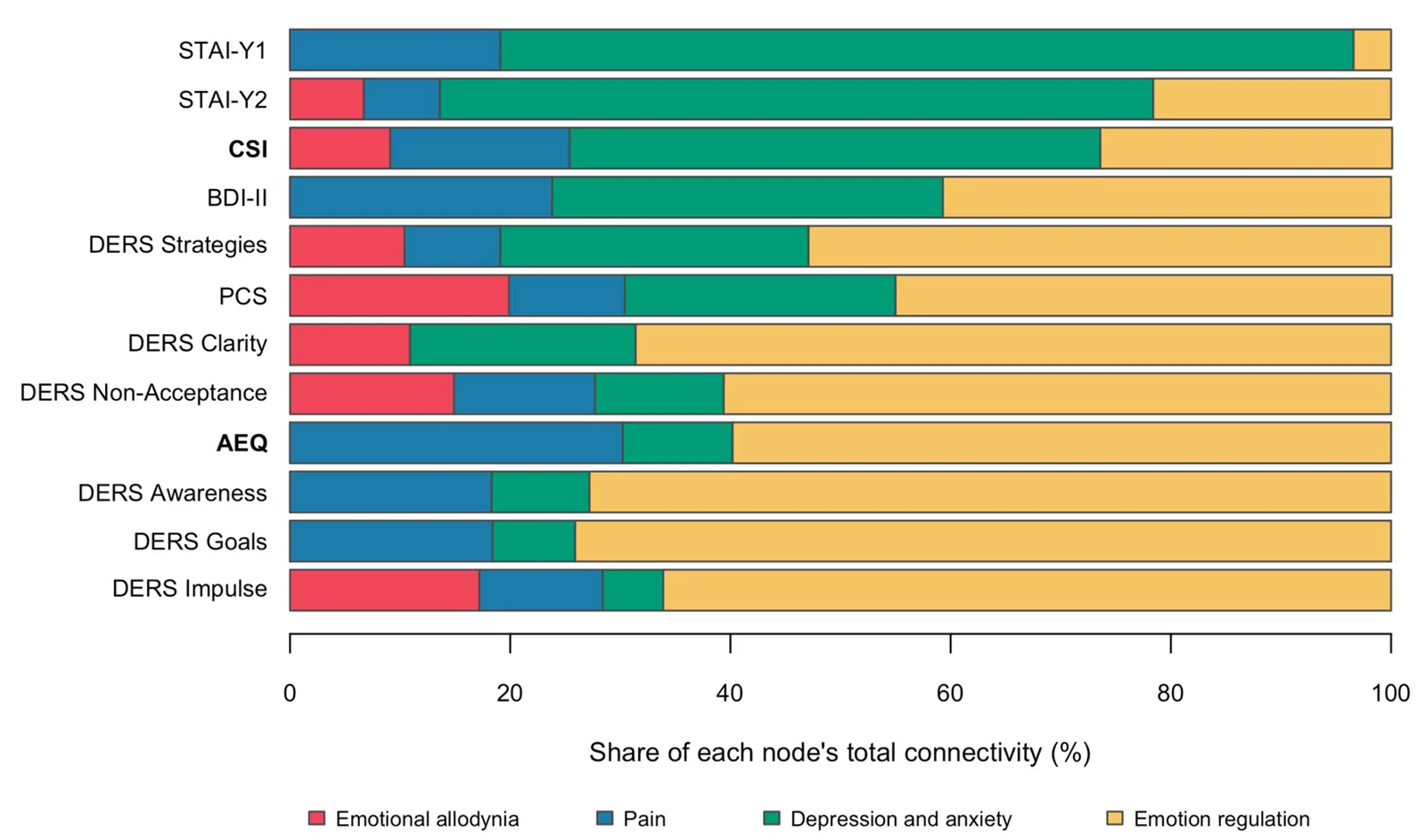
Share of each node’s total connectivity directed at each of the four domains, ordered by the share directed at depression and anxiety. Bars sum to 100% and are computed from absolute edge weights, so a negative edge contributes as a positive edge of the same magnitude does. For a node belonging to a domain with more than one member, the corresponding segment represents its within-domain connectivity. Two nodes, shown in bold, are discussed in the text. The AEQ directs 59.8% of its connectivity to emotion regulation and 30.2% to the pain domain, and only 10.0% to depression and anxiety. Of all nodes lying outside the emotion-regulation domain and of all nodes lying outside the pain domain, it is in both cases the one directing the largest share to that domain. The CSI, an instrument of the pain domain, directs 48.2% of its connectivity to depression and anxiety. AEQ—Emotional Allodynia Questionnaire; PCS—Pain Catastrophizing Scale; CSI—Central Sensitization Inventory; BDI-II—Beck Depression Inventory-II; STAI-Y1/Y2—state and trait anxiety; DERS—Difficulties in Emotion Regulation Scale.

**Table 1:** Demographic, clinical and interpersonal characteristics of the cohort (*n* = 149).

Variable	Overall (*n* = 149)
**Demographics**	
Age, years, mean (SD)	57.51 (10.89)
Female sex, *n* (%)	137 (91.9)
Level of education, *n* (%)	
Primary school	14 (9.4)
Middle school	65 (43.6)
High school	53 (35.6)
University degree	17 (11.4)
Occupational status, *n* (%)	
Employed	45 (30.2)
Unemployed	80 (53.7)
Retired	24 (16.1)
Recognized disability, *n* (%)	96 (64.4)
**Pain characteristics**	
Duration of index pain, *n* (%)	
Between 6 months and 1 year	4 (2.7)
Between 1 and 3 years	18 (12.1)
>3 to 5 years	20 (13.4)
>5 to 10 years	30 (20.1)
>10 years	77 (51.7)
Current pain intensity (NRS 0–10), median (IQR)	8 (3)
Current pain intensity (NRS 0–10), mean (SD)	7.93 (1.75)
**Psychiatric comorbidity, documented**	
Depressive disorder, *n* (%)	68 (45.6)
Panic disorder, *n* (%)	2 (1.3)
No psychiatric comorbidity, *n* (%)	53 (35.6)
Current psychotherapy, *n* (%)	23 (15.4)
**Medication, self-reported current therapy**	
Any recorded drug class, *n* (%)	134 (89.9)
Gabapentinoids, *n* (%)	54 (36.2)
Antidepressants, *n* (%)	58 (38.9)
SNRI	26 (17.4)
SSRI	10 (6.7)
Tricyclics	21 (14.1)
Other antidepressants	8 (5.4)
Opioids, *n* (%)	54 (36.2)
NSAIDs, *n* (%)	33 (22.1)
Paracetamol, *n* (%)	26 (17.4)
Medical cannabis, *n* (%)	19 (12.8)
Muscle relaxants, *n* (%)	31 (20.8)
Corticosteroids, *n* (%)	14 (9.4)
Benzodiazepines, *n* (%)	18 (12.1)
Hypnotics, *n* (%)	4 (2.7)
Antipsychotics, *n* (%)	2 (1.3)
Mood stabilizers, *n* (%)	4 (2.7)
Psychotropic medication (self-declared), *n* (%)	81 (54.4)
**Lifestyle and self-rated outcomes**	
Physical activity level, *n* (%)	
Sedentary (less than 1 h/week)	90 (60.4)
Light (1 to 2 h/week)	38 (25.5)
Moderate (3 to 5 h/week)	19 (12.8)
Vigorous (6 h/week or more)	2 (1.3)
Subjective sleep quality (0–10), mean (SD)	4.46 (2.61)
Subjective quality of life (0–10), mean (SD)	4.66 (2.20)
**Interpersonal context**	
Lives alone, *n* (%)	24 (16.1)
Current romantic relationship, *n* (%)	118 (79.2)
Lasting more than 3 years (*n* = 118)	114 (96.6)
Not in a relationship, *n* (%)	31 (20.8)
Single for more than 3 years (*n* = 31)	28 (90.3)
Significant breakup in the last 3 years, *n* (%)	23 (15.4)
Still emotionally involved (*n* = 23)	17 (73.9)
Someone to rely on in difficult times, *n* (%)	120 (80.5)
Perceived emotional support (0–10), mean (SD)	5.94 (3.23)
History of abandonment, betrayal or rejection, *n* (%)	
Never	61 (40.9)
Once	35 (23.5)
Multiple times	53 (35.6)
Tendency to form intense attachments rapidly, *n* (%)	63 (42.3)
Distress at perceived interpersonal distance, *n* (%)	117 (78.5)

Values are *n* (%) unless otherwise stated. Percentages are computed over the full cohort, except in the indented rows that carry their own denominator (*n* = …). Education, occupational status and current pharmacological treatment were recorded as free text and coded into the categories shown; drug classes, including the antidepressant subclasses, are not mutually exclusive and therefore sum to more than the rows above them. Duration of index pain refers to the pain the patient identified as currently most distressing, not to the time since the diagnosis of fibromyalgia. Depressive and panic disorders are those documented at enrolment; they are not mutually exclusive and do not account for every patient with a documented comorbidity, the remainder having recorded anxiety symptoms without either diagnosis. Perceived emotional support and the subjective sleep and quality-of-life ratings were single items scored 0–10, with higher values indicating a better state. IQR—interquartile range; NRS—numerical rating scale; NSAID—non-steroidal anti-inflammatory drug; SD—standard deviation; SNRI—serotonin–noradrenaline reuptake inhibitor; SSRI—selective serotonin reuptake inhibitor.

**Table 2:** Descriptive statistics of the twelve network nodes (*n* = 149).

Node	M	SD	Mdn	Q25	Q75	Min	Max	Skew
AEQ	18.09	10.06	17	11	25	1	41	0.15
PCS	31.78	11.70	33	22	41	4	52	−0.29
CSI	60.95	15.66	61	50	73	24	92	−0.22
BDI-II	22.18	10.39	20	15	29	3	53	0.55
STAI-Y1	49.09	13.29	48	39	60	20	79	0.04
STAI-Y2	47.95	9.55	47	41	56	26	71	0.10
DERS Non-Acceptance	14.03	6.51	13	8	19	6	30	0.51
DERS Goals	15.01	5.16	15	11	19	5	25	−0.01
DERS Strategies	18.81	7.39	18	12	24	8	38	0.35
DERS Impulse	12.11	5.09	11	8	15	6	30	1.02
DERS Clarity	11.09	4.41	11	7	14	5	25	0.63
DERS Awareness	6.84	3.50	6	3	10	3	15	0.59

M—mean; SD—standard deviation; Mdn—median; Q25 and Q75—first and third quartiles; Min and Max—observed minimum and maximum; Skew—skewness. AEQ—Emotional Allodynia Questionnaire; PCS—Pain Catastrophizing Scale; CSI—Central Sensitization Inventory; BDI-II—Beck Depression Inventory-II; STAI-Y1/Y2—State–Trait Anxiety Inventory—state and trait forms; DERS—Difficulties in Emotion Regulation Scale.

**Table 3:** Zero-order (Spearman ρ) and regularized partial associations of the AEQ across three model specifications.

Node	Zero-Order ρ	Network Edge (γ = 0.5)	Thresholded	γ = 0.25
PCS	0.570	0.190	0.185	0.190
DERS Impulse	0.595	0.184	0.184	0.184
DERS Strategies	0.580	0.124	0	0.124
DERS Non-Acceptance	0.463	0.102	0	0.102
STAI-Y2	0.566	0.082	0	0.082
DERS Clarity	0.444	0.078	0	0.078
CSI	0.441	0.056	0	0.056
BDI-II	0.502	0	0	0
STAI-Y1	0.506	0	0	0
DERS Goals	0.439	0	0	0
DERS Awareness	0.049 †	0	0	0

All zero-order correlations *p* < 0.001 except † (*p* = 0.556). AEQ—Emotional Allodynia Questionnaire; PCS—Pain Catastrophizing Scale; CSI—Central Sensitization Inventory; BDI-II—Beck Depression Inventory-II; STAI-Y1/Y2—State–Trait Anxiety Inventory, state and trait forms; DERS—Difficulties in Emotion Regulation Scale.

**Table 4:** Regularized partial correlations (network edges) of the AEQ and of the PCS with the remaining ten nodes, by community ( 149).

Node	Community	AEQ Edge	PCS Edge
CSI	Pain	0.056	0.101
BDI-II	Depression and anxiety	0	0.114
STAI-Y1 (state)	Depression and anxiety	0	0.121
STAI-Y2 (trait)	Depression and anxiety	0.082	0
DERS Non-Acceptance	Emotion regulation	0.102	0.088
DERS Goals	Emotion regulation	0	0
DERS Strategies	Emotion regulation	0.124	0.102
DERS Impulse	Emotion regulation	0.184	0.119
DERS Clarity	Emotion regulation	0.078	0
DERS Awareness	Emotion regulation	0	−0.121

Community is the a priori domain to which each node was assigned for the bridge-centrality analysis (Section 2.5). The AEQ–PCS edge itself was 0.190. AEQ—Emotional Allodynia Questionnaire; PCS—Pain Catastrophizing Scale; CSI—Central Sensitization Inventory; BDI-II—Beck Depression Inventory-II; STAI-Y1/Y2—State–Trait Anxiety Inventory, state and trait forms; DERS—Difficulties in Emotion Regulation Scale.

## Data Availability

The Spearman correlation matrix of the twelve nodes, from which the network, its centrality and bridge indices, the domain-connectivity proportions and the γ = 0.25 solution can be reproduced exactly, is available from the corresponding author on reasonable request, together with the analysis scripts. The remaining analyses reported here require item-level or case-level data and cannot be reproduced from that matrix: internal consistency, node predictability, the redundancy procedure, the nonparanormal and subscale-level re-estimations, and all bootstrapping. Individual-level data are likewise available on reasonable request, within the limits of the ethical approval.

## Supplementary Materials

The following supporting information can be downloaded at: https://www.mdpi.com/article/10.3390/medsci14060599/s1, Table S1, internal consistency of the twelve network nodes in the analyzed sample (*n* = 149); Table S2, full weighted adjacency matrix of the regularized partial correlation network (EBICglasso, γ = 0.5, Spearman correlations; *n* = 149); Table S3, node predictability and centrality indices; Table S4, bridge strength of the twelve nodes with respect to the four a priori communities, with bootstrap percentile intervals (2.5th–97.5th; *n* = 149; 5000 non-parametric replications); Table S5, proportion of each node’s total connectivity directed at each domain, with bootstrap percentile intervals (*n* = 149; 5000 non-parametric replications); Table S6, zero-order Spearman correlation matrix of the twelve nodes (*n* = 149); Table S7, bootstrap edge inclusion proportions for the AEQ (*n* = 149); Figure S1a, bootstrapped 95% confidence intervals of the 66 edge weights (5000 non-parametric replications), ordered by the sample estimate; Figure S1b, edge-difference test (5000 replications), restricted to the 43 non-zero edges and ordered by edge weight; Figure S1c, strength-difference test (5000 replications), with nodes ordered by strength centrality; Table S8, goldbricker redundancy analysis (*n* = 149); Table S9, partial Spearman correlations of the AEQ and of the PCS with the remaining nodes, each adjusted for the other (*n* = 149); Table S10, edge weights of the network re-estimated with threshold = TRUE, which enforces higher specificity at the cost of sensitivity; Table S11, edge weights of the network re-estimated with the EBIC hyperparameter set to γ = 0.25, the value used by Oliva et al., instead of the more conservative γ = 0.5 adopted here (*n* = 149); Table S12, sensitivity analysis: edges of the AEQ under the nonparanormal transformation; Table S13, exploratory factor structure of the twelve nodes (*n* = 149, Spearman correlation matrix); Table S14, sensitivity to node granularity: edges of the AEQ in a network in which the Pain Catastrophizing Scale is entered as its three subscales rather than as a total score (fourteen nodes; *n* = 149; EBICglasso, γ = 0.5, Spearman); Table S15, node predictability re-estimated after removing state anxiety from the model (*n* = 149; mixed graphical model, all nodes Gaussian, EBIC with γ = 0.5); Table S16, sensitivity to node granularity in both directions: the proportion of the AEQ’s total connectivity directed at each domain under four representations of the same data; Table S17, attenuation of the association between the AEQ and the BDI-II across a nested sequence of adjustment sets (*n* = 149); Table S18, evidence bearing on the absence of a conditional association between the AEQ and the BDI-II, none of which relies on LASSO selection (*n* = 149); Table S19, characteristics of the three reports from the PEARL cohort; Table S20, association between the AEQ and the BDI-II adjusted for, and stratified by, documented depressive disorder and current antidepressant use (*n* = 149); Table S21, agreement between the predictors of the nodewise predictability models and the edges of the network (*n* = 149).

## Abbreviations

AEQ: Emotional Allodynia Questionnaire; BDI-II: Beck Depression Inventory-II; CS: correlation stability; CSI: Central Sensitization Inventory; DERS: Difficulties in Emotion Regulation Scale; EBIC: Extended Bayesian Information Criterion; GGM: Gaussian graphical model; LASSO: least absolute shrinkage and selection operator; PCS: Pain Catastrophizing Scale; STAI-Y1/Y2: State–Trait Anxiety Inventory, state and trait forms.

## References

[1] Kosek E., Clauw D., Nijs J., Baron R., Gilron I., Harris R.E., Mico J.-A., Rice A.S.C., Sterling M. (2021). Chronic Nociplastic Pain Affecting the Musculoskeletal System: Clinical Criteria and Grading System. Pain.

[2] Williams D.A., Clauw D.J. (2026). Fibromyalgia. N. Engl. J. Med..

[3] Lumley M.A., Schubiner H., Lockhart N.A., Kidwell K.M., Harte S.E., Clauw D.J., Williams D.A. (2017). Emotional Awareness and Expression Therapy, Cognitive Behavioral Therapy, and Education for Fibromyalgia: A Cluster-Randomized Controlled Trial. Pain.

[4] Lumley M.A., Schubiner H. (2019). Emotional Awareness and Expression Therapy for Chronic Pain: Rationale, Principles and Techniques, Evidence, and Critical Review. Curr. Rheumatol. Rep..

[5] Pinto A.M., Geenen R., Wager T.D., Lumley M.A., Häuser W., Kosek E., Ablin J.N., Amris K., Branco J., Buskila D. (2023). Emotion Regulation and the Salience Network: A Hypothetical Integrative Model of Fibromyalgia. Nat. Rev. Rheumatol..

[6] Ushinsky A., Reinhardt L.E., Simmons A.N., Strigo I.A. (2013). Further Evidence of Emotional Allodynia in Unmedicated Young Adults with Major Depressive Disorder. PLoS ONE.

[7] Corriero A., Giglio M., Pilolla A., Galdini F., Mucci O., Vurro M., Fornarelli F., Di Venosa C., Trerotoli P., Puntillo F. (2026). The Emotional Allodynia Questionnaire: Preliminary Validation and Clinical Phenotyping in Fibromyalgia. J. Pain Res..

[8] Corriero A., Giglio M., Pilolla A., Galdini F., Fornarelli F., Di Venosa C., Costantino E., De Orsi D., Varrassi G., Trerotoli P. (2026). Which Facets of Emotion Dysregulation Are Uniquely Associated with Emotional Allodynia in Fibromyalgia? A Preliminary Analysis Using the Emotional Allodynia Questionnaire and the Difficulties in Emotion Regulation Scale. Front. Psychol..

[9] Epskamp S., Fried E.I. (2018). A Tutorial on Regularized Partial Correlation Networks. Psychol. Methods.

[10] Jones P.J., Ma R., McNally R.J. (2021). Bridge Centrality: A Network Approach to Understanding Comorbidity. Multivar. Behav. Res..

[11] Varol U., Úbeda-D’Ocasar E., Cigarán-Méndez M., Arias-Buría J.L., Fernández-de-Las-Peñas C., Gallego-Sendarrubias G.M., Valera-Calero J.A. (2023). Understanding the Psychophysiological and Sensitization Mechanisms Behind Fibromyalgia Syndrome: A Network Analysis Approach. Pain Med..

[12] Valera-Calero J.A., Arendt-Nielsen L., Cigarán-Méndez M., Fernández-de-las-Peñas C., Varol U. (2022). Network Analysis for Better Understanding the Complex Psycho-Biological Mechanisms behind Fibromyalgia Syndrome. Diagnostics.

[13] Pacho-Hernández J.C., Fernández-Palacios F.G., Tejera-Alonso Á., Fernández-de-las-Peñas C., Varol U., Valera-Calero J.A., Fernández-Méndez L.M., Cigarán-Mendez M. (2024). Understanding the Associations between Executive Function and Psychological Variables in Fibromyalgia Syndrome: A Network Analysis Approach. Healthcare.

[14] Aghbelaghi D.T., Jalali M., Tayim N., Kiyani R. (2025). A Network Analysis of Depression and Cognitive Impairments in Fibromyalgia: A Secondary Analysis Study. Psychiatr. Q..

[15] Malka T., Marom-Harel H., Frumer L., Agmon-Levin N., Taub R., Glick I., Admon R., Hanuka S., Peretz-Tamari N., Brown A. (2025). Fibromyalgia and Depression: A Network Analysis Approach. Behav. Ther..

[16] Reichert J., Drusko A., Beiner E., Hermes M., Papaioannou V., Häuser W., Eich W., Tesarz J. (2026). Symptom-Level Comorbidity of Fibromyalgia and Depression: A Network Approach within a Large Clinical Sample. Psychother. Psychosom..

[17] Oliva F., Merola M., Olivero A., Leombruni P. (2026). The Relationship Between Pain and Depression in Fibromyalgia: Structural Equation Modeling and Network Analysis. Int. J. Environ. Res. Public Health.

[18] Wolfe F., Clauw D.J., Fitzcharles M.-A., Goldenberg D.L., Häuser W., Katz R.L., Mease P.J., Russell A.S., Russell I.J., Walitt B. (2016). 2016 Revisions to the 2010/2011 Fibromyalgia Diagnostic Criteria. Semin. Arthritis Rheum..

[19] Sullivan M.J.L., Bishop S.R., Pivik J. (1995). The Pain Catastrophizing Scale: Development and Validation. Psychol. Assess..

[20] Mayer T.G., Neblett R., Cohen H., Howard K.J., Choi Y.H., Williams M.J., Perez Y., Gatchel R.J. (2012). The Development and Psychometric Validation of the Central Sensitization Inventory. Pain Pract..

[21] Beck A.T., Steer R.A., Brown G.K. (1996). Manual for the Beck Depression Inventory–II.

[22] Spielberger C.D., Gorsuch R.L., Lushene R., Vagg P.R., Jacobs G.A. (1983). Manual for the State-Trait Anxiety Inventory (Form Y).

[23] Gratz K.L., Roemer L. (2004). Multidimensional Assessment of Emotion Regulation and Dysregulation: Development, Factor Structure, and Initial Validation of the Difficulties in Emotion Regulation Scale. J. Psychopathol. Behav. Assess..

[24] Sighinolfi C., Norcini Pala A., Chiri L., Marchetti I., Sica C. (2010). Difficulties in Emotion Regulation Scale (DERS): Traduzione e Adattamento Italiano. Psicoter. Cogn. E Comport..

[25] Haslbeck J.M.B., Waldorp L.J. (2018). How Well Do Network Models Predict Observations? On the Importance of Predictability in Network Models. Behav. Res..

[26] Epskamp S., Borsboom D., Fried E.I. (2018). Estimating Psychological Networks and Their Accuracy: A Tutorial Paper. Behav. Res..

[27] Jones P. (2025). Networktools: Tools for Identifying Important Nodes in Networks, R Package Version 1.6.0. https://CRAN.R-project.org/package=networktools.

[28] R Core Team (2025). R: A Language and Environment for Statistical Computing.

[29] Burger J., Isvoranu A.-M., Lunansky G., Haslbeck J.M.B., Epskamp S., Hoekstra R.H.A., Fried E.I., Borsboom D., Blanken T.F. (2023). Reporting Standards for Psychological Network Analyses in Cross-Sectional Data. Psychol. Methods.

[30] Adams G.R., Gandhi W., Harrison R., van Reekum C.M., Wood-Anderson D., Gilron I., Salomons T.V. (2023). Do “Central Sensitization” Questionnaires Reflect Measures of Nociceptive Sensitization or Psychological Constructs? A Systematic Review and Meta-Analyses. Pain.

[31] Giesecke T., Williams D.A., Harris R.E., Cupps T.R., Tian X., Tian T.X., Gracely R.H., Clauw D.J. (2003). Subgrouping of Fibromyalgia Patients on the Basis of Pressure-Pain Thresholds and Psychological Factors. Arthritis Rheum..

[32] Riquelme-Aguado V., González-Álvarez M.E., Di-Bonaventura S., Rodríguez-Lagos L., Zabarte del Campo A., Gómez-Esquer F., Díaz-Gil G., Gil-Crujera A. (2026). Associations Between Genetic Polymorphisms and Psychological Variables in Women with Fibromyalgia: A Cross-Sectional Study. Eur. J. Pain.

