# Supplementary material for: Is Emotional Allodynia in Fibromyalgia Reducible to Depression? A Preliminary Network Analysis in 149 Patients
Source: Med Sci (Basel). 2026 Sep 23;14(6):599. doi: 10.3390/medsci14060599 (PMC13707321; doi:10.3390/medsci14060599)
Supplement: Supplementary file 1 [file medsci-14-00599-s001.zip › medsci-4565531-supplementary.pdf]

Supplementary Materials

Is Emotional Allodynia in Fibromyalgia Reducible to Depression? A Preliminary Network Analysis in 149 Patients

Alberto Corriero [1,*], Mariateresa Giglio [1], Alfonso Pilolla [1], Filomena Galdini [1], Cinzia Di Venosa [1], Elisabetta Costantino [2], Daniela De Orsi [3], Giustino Varrassi [4], Paolo Trerotoli [5] and Filomena Puntillo [1]

[1] Pain and ICU Section, Department of Interdisciplinary Medicine, University of Bari Aldo Moro, Piazza G. Cesare 11, 70124 Bari, Italy; mariateresa.giglio@uniba.it (M.G.); alfonso.pilolla@gmail.com (A.P.); milena200392@gmail.com (F.G.); cinziadivenosa@gmail.com (C.D.V.); filomena.puntillo@uniba.it (F.P.)
[2] Nutrimente Onlus, 20121 Milano, Italy; elisabettacostantinopsi@gmail.com
[3] Centro di Salute Mentale Area 5, ASL Bari, 70124 Bari, Italy; daniela.deorsi@asl.bari.it
[4] Paolo Procacci Foundation, 00193 Rome, Italy; giuvarr@gmail.com
[5] Statistics Section, Department of Interdisciplinary Medicine, University of Bari Aldo Moro, Piazza G. Cesare 11, 70124 Bari, Italy; paolo.trerotoli@uniba.it
* Correspondence: alberto.corriero@gmail.com

Table S1. Internal consistency of the twelve network nodes in the analyzed sample (n = 149). Item-level responses were scored with the same procedure used to derive the node scores, including reverse-keyed items. DERS subscale membership was recovered empirically by identifying, for each subscale, the item subset whose sum reproduced the stored subscale score exactly for every participant. Reliability is acceptable to excellent for eleven nodes; DERS Clarity falls below the conventional 0.70 threshold and its edges should be read with that in mind.

Node	Items	α	ω total
**AEQ**	11	0.909	0.909
**PCS**	13	0.901	0.907
**CSI**	25	0.859	0.866
**BDI-II**	21	0.878	0.889
**STAI-Y1**	20	0.938	0.940
**STAI-Y2**	20	0.915	0.918
**DERS Non-Acceptance**	6	0.849	0.854
**DERS Goals**	5	0.756	0.765
**DERS Strategies**	8	0.813	0.819
**DERS Impulse**	6	0.780	0.804
**DERS Clarity**	5	0.661	0.670
**DERS Awareness**	3	0.747	0.760

Note: α—Cronbach's alpha; ω—McDonald's omega total. AEQ—Emotional Allodynia Questionnaire; PCS—Pain Catastrophizing Scale; CSI—Central Sensitization Inventory; BDI-II—Beck Depression Inventory-II; STAI-Y1/Y2—State–Trait Anxiety Inventory, state and trait forms; DERS—Difficulties in Emotion Regulation Scale.

Table S2. Full weighted adjacency matrix of the regularized partial correlation network (EBICglasso, γ = 0.5, Spearman correlations; n = 149). Values are regularized partial correlations; dashes denote edges shrunk to exactly zero.

	AEQ	PCS	CSI	BDI-II	S-Anx	T-Anx	NonAc	Goals	Strat	Impul	Clar	Aware
AEQ	—	0.190	0.056	—	—	0.082	0.102	—	0.124	0.184	0.078	—
PCS	0.190	—	0.101	0.114	0.121	—	0.088	—	0.102	0.119	—	-0.121

CSI	0.056	0.101	—	0.146	0.067	0.084	—	0.163	—	—	—	—
BDI-II	—	0.114	0.146	—	0.180	0.208	0.021	0.067	0.185	—	0.112	0.059
S-Anx	—	0.121	0.067	0.180	—	0.583	—	—	0.030	—	0.003	—
T-Anx	0.082	—	0.084	0.208	0.583	—	0.060	—	0.116	0.058	0.030	—
NonAc	0.102	0.088	—	0.021	—	0.060	—	0.166	—	0.183	—	-0.068
Goals	—	—	0.163	0.067	—	—	0.166	—	0.223	0.193	—	-0.078
Strat	0.124	0.102	—	0.185	0.030	0.116	—	0.223	—	0.137	0.113	0.152
Impul	0.184	0.119	—	—	—	0.058	0.183	0.193	0.137	—	0.191	—
Clar	0.078	—	—	0.112	0.003	0.030	—	—	0.113	0.191	—	0.184
Aware	—	-0.121	—	0.059	—	—	-0.068	-0.078	0.152	—	0.184	—

Note: AEQ—Emotional Allodynia Questionnaire; PCS—Pain Catastrophizing Scale; CSI—Central Sensitization Inventory; BDI-II—Beck Depression Inventory-II; S-Anx—STAI-Y1 (state anxiety); T-Anx—STAI-Y2 (trait anxiety); NonAc, Goals, Strat, Impul, Clar and Aware—the six DERS facets (Non-Acceptance, Goals, Strategies, Impulse, Clarity, Awareness).

Table S3. Node predictability and centrality indices. R^2 is the proportion of variance in each node explained by all its neighbours (mixed graphical model, all nodes Gaussian, EBIC neighbourhood selection with lambdaGam = 0.5; n = 149). Nodes are ordered by strength centrality.

Node	R^2	Strength	Strength (z)
STAI-Y2	0.693	1.222	1.50
DERS Strategies	0.467	1.182	1.32
BDI-II	0.526	1.092	0.88
DERS Impulse	0.574	1.065	0.75
STAI-Y1	0.668	0.984	0.37
PCS	0.436	0.956	0.23
DERS Goals	0.496	0.889	-0.09
AEQ	0.402	0.815	-0.44
DERS Clarity	0.405	0.711	-0.94
DERS Non-Acceptance	0.197	0.688	-1.05
DERS Awareness	0.058	0.663	-1.17
CSI	0.415	0.618	-1.38

Note: Strength (z) is strength centrality expressed as a z-score across nodes. Closeness and betweenness were computed but are not reported, being markedly less stable in cross-sectional networks and of questionable suitability for networks of this kind. AEQ—Emotional Allodynia Questionnaire; PCS—Pain Catastrophizing Scale; CSI—Central Sensitization Inventory; BDI-II—Beck Depression Inventory-II; STAI-Y1/Y2—State–Trait Anxiety Inventory, state and trait forms; DERS—Difficulties in Emotion Regulation Scale.

Table S4. Bridge strength of the twelve nodes with respect to the four a priori communities, with bootstrap percentile intervals (2.5th–97.5th; n = 149; 5000 non-parametric replications). Values are reported descriptively and are not ranked against one another: because the AEQ forms a community of one, all of its edges are between-community and its bridge strength equals its total strength, so its value is not comparable with that of nodes that lose their within-community edges.

Node	Bridge strength	Percentile interval
PCS	0.856	[0.635, 1.082]
**AEQ**	**0.815**	**[0.657, 1.051]**
BDI-II	0.704	[0.482, 1.122]
DERS Strategies	0.557	[0.376, 0.752]
CSI	0.517	[0.382, 0.835]
STAI-Y2	0.430	[0.260, 0.661]
DERS Impulse	0.361	[0.201, 0.652]
DERS Non-Acceptance	0.271	[0.100, 0.504]

DERS Goals	0.230	[0.096, 0.465]
DERS Clarity	0.223	[0.082, 0.491]
STAI-Y1	0.221	[0.100, 0.480]
DERS Awareness	0.180	[0.010, 0.487]

Note: AEQ—Emotional Allodynia Questionnaire; PCS—Pain Catastrophizing Scale; CSI—Central Sensitization Inventory; BDI-II—Beck Depression Inventory-II; S-Anx—STAI-Y1 (state anxiety); T-Anx—STAI-Y2 (trait anxiety); NonAc, Goals, Strat, Impul, Clar and Aware—the six DERS facets (Non-Acceptance, Goals, Strategies, Impulse, Clarity, and Awareness).

Table S5. Proportion of each node's total connectivity directed at each domain, with bootstrap percentile intervals (n = 149; 5000 non-parametric replications). These are the values plotted in Figure 2 of the main text; the total strength of each node, which is the denominator of the proportion, is given in Table S3. For each node, the proportion is the sum of the absolute weights of its edges directed at nodes of the given domain, divided by the sum of the absolute weights of all its edges, expressed as a percentage; the four proportions of a row sum to 100%. Intervals are the 2.5th and 97.5th percentiles of the same bootstrap distribution used for the edge weights, and are wide: the proportions are descriptive of the estimated network and differences between nodes should not be treated as established. The dash marks the AEQ's own domain, which has no other member. The four domains contain one, two, three and six nodes, respectively, so the shares a given node directs at different domains reflect domain size as well as connectivity; the sensitivity analysis of Table S14 addresses this. Weights are taken in absolute value, so a negative edge contributes to a share exactly as a positive edge of the same magnitude does: the 18.3% that DERS Awareness directs at the pain domain is its single negative edge with the PCS.

**Node**	**Emotional allodynia**	**Pain**	**Depression and anxiety**	**Emotion regulation**
**AEQ**	—	30.2 [7.3, 51.5]	10.0 [0.0, 28.4]	59.8 [35.5, 82.6]
**PCS**	19.9 [3.3, 36.0]	10.5 [0.0, 24.5]	24.6 [10.9, 43.5]	45.1 [23.5, 66.0]
**CSI**	9.1 [0.0, 28.5]	16.3 [0.0, 35.2]	48.2 [19.3, 71.9]	26.5 [8.7, 58.9]
**BDI-II**	0.0 [0.0, 8.8]	23.8 [6.6, 39.4]	35.5 [20.0, 50.3]	40.7 [23.4, 59.4]
**STAI-Y1**	0.0 [0.0, 9.1]	19.1 [2.1, 30.6]	77.5 [58.3, 88.9]	3.4 [0.0, 23.2]
**STAI-Y2**	6.7 [0.0, 15.3]	6.9 [0.0, 20.8]	64.8 [50.6, 76.2]	21.6 [9.9, 34.6]
**DERS Non-Acceptance**	14.9 [0.0, 33.4]	12.8 [0.0, 35.8]	11.7 [0.0, 30.7]	60.6 [34.9, 84.5]
**DERS Goals**	0.0 [0.0, 9.8]	18.4 [1.9, 33.3]	7.5 [0.0, 26.9]	74.1 [50.9, 87.8]
**DERS Strategies**	10.4 [0.0, 24.2]	8.7 [0.0, 20.7]	28.0 [13.1, 43.4]	52.9 [36.0, 68.5]
**DERS Impulse**	17.2 [4.3, 28.6]	11.2 [0.0, 28.9]	5.5 [0.0, 20.1]	66.1 [45.5, 80.5]
**DERS Clarity**	10.9 [0.0, 27.8]	0.0 [0.0, 17.8]	20.5 [4.6, 39.3]	68.6 [42.7, 88.1]
**DERS Awareness**	0.0 [0.0, 14.5]	18.3 [0.0, 37.2]	8.9 [0.0, 28.7]	72.8 [44.8, 98.3]

Note: AEQ—Emotional Allodynia Questionnaire; PCS—Pain Catastrophizing Scale; CSI—Central Sensitization Inventory; BDI-II—Beck Depression Inventory-II; STAI-Y1/Y2—State–Trait Anxiety Inventory, state and trait forms; DERS—Difficulties in Emotion Regulation Scale.

Table S6. Zero-order Spearman correlation matrix of the twelve nodes (n = 149). Comparison with Table S2 shows which bivariate associations do not survive conditioning on the remaining variables.

	**AEQ**	**PCS**	**CSI**	**BDI-II**	**S-Anx**	**T-Anx**	**NonAc**	**Goals**	**Strat**	**Impul**	**Clar**	**Aware**
AEQ	1.000	0.570	0.441	0.502	0.506	0.566	0.463	0.439	0.580	0.595	0.444	0.049
PCS	0.570	1.000	0.487	0.589	0.590	0.584	0.458	0.437	0.573	0.560	0.358	-0.037
CSI	0.441	0.487	1.000	0.570	0.548	0.565	0.342	0.488	0.468	0.431	0.272	0.074
BDI-II	0.502	0.589	0.570	1.000	0.737	0.760	0.441	0.535	0.686	0.482	0.506	0.217

S-Anx	0.506	0.590	0.548	0.737	1.000	0.869	0.385	0.473	0.631	0.508	0.447	0.115
T-Anx	0.566	0.584	0.565	0.760	0.869	1.000	0.468	0.518	0.675	0.584	0.480	0.136
NonAc	0.463	0.458	0.342	0.441	0.385	0.468	1.000	0.488	0.444	0.533	0.329	-0.048
Goals	0.439	0.437	0.488	0.535	0.473	0.518	0.488	1.000	0.601	0.581	0.332	-0.006
Strat	0.580	0.573	0.468	0.686	0.631	0.675	0.444	0.601	1.000	0.630	0.527	0.265
Impul	0.595	0.560	0.431	0.482	0.508	0.584	0.533	0.581	0.630	1.000	0.522	0.096
Clar	0.444	0.358	0.272	0.506	0.447	0.480	0.329	0.332	0.527	0.522	1.000	0.302
Aware	0.049	-0.037	0.074	0.217	0.115	0.136	-0.048	-0.006	0.265	0.096	0.302	1.000

Note: AEQ—Emotional Allodynia Questionnaire; PCS— Pain Catastrophizing Scale; CSI—Central Sensitization Inventory; BDI-II—Beck Depression Inventory-II; S-Anx—STAI-Y1 (state anxiety); T-Anx—STAI-Y2 (trait anxiety); NonAc, Goals, Strat, Impul, Clar and Aware—the six DERS facets (Non-Acceptance, Goals, Strategies, Impulse, Clarity, and Awareness).

Table S7. Bootstrap edge inclusion proportions for the AEQ (n = 149): the percentage of 5000 bootstrap samples in which each of the eleven edges of the AEQ was estimated as non-zero. These proportions, and the bridge-strength intervals of Table S4, were computed from the additional bootstrap described in Section 2.6, in which the network was re-estimated in each resample; Figures S1a-c derive from the separate bootnet resampling of the same size. Both used 5000 replications, all of which converged, so the two differ only by Monte Carlo variation, of the order of one percentage point on these proportions. The separation between the seven non-zero edges and the four zero edges holds under either.

**Node**	**Edge weight with the AEQ**	**Inclusion (%)**
DERS Impulse	0.184	99.6
PCS	0.190	98.9
DERS Strategies	0.124	91.2
DERS Non-Acceptance	0.102	86.6
STAI-Y2	0.082	85.7
DERS Clarity	0.078	80.4
CSI	0.056	74.0
DERS Awareness	0 (absent)	35.3
DERS Goals	0 (absent)	29.4
STAI-Y1	0 (absent)	27.8
BDI-II	0 (absent)	26.0

Note: AEQ—Emotional Allodynia Questionnaire; PCS—Pain Catastrophizing Scale; CSI—Central Sensitization Inventory; BDI-II—Beck Depression Inventory-II; STAI-Y1/Y2—State–Trait Anxiety Inventory—state and trait forms; DERS—Difficulties in Emotion Regulation Scale.

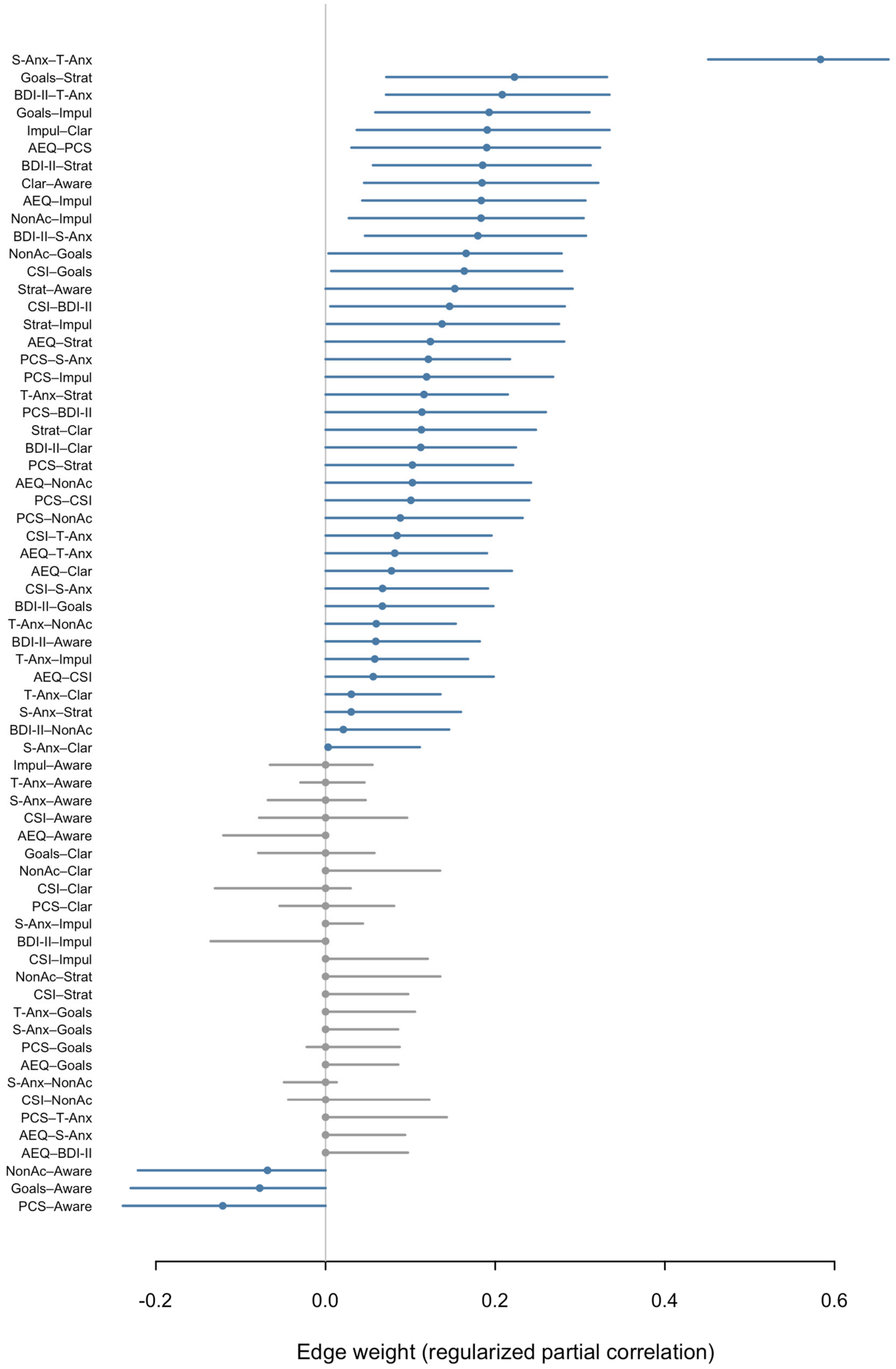

Figure S1a. Bootstrapped 95% confidence intervals of the 66 edge weights (5000 non-parametric replications), ordered by the sample estimate. Points are the sample estimates and bars the 2.5th–97.5th percentiles of the bootstrap distribution. Edges estimated as non-zero in the sample network are shown in blue; edges shrunk to zero are shown in grey. Abbreviations: AEQ—Emotional Allodynia Questionnaire; PCS—Pain Catastrophizing Scale; CSI—Central Sensitization Inventory; BDI-II—Beck Depression Inventory-II; S-Anx and T-Anx—STAI-Y1 (state) and STAI-Y2 (trait) anxiety;

NonAc, Goals, Strat, Impul, Clar and Aware—the Non-Acceptance, Goals, Strategies, Impulse, Clarity and Awareness subscales of the DERS.

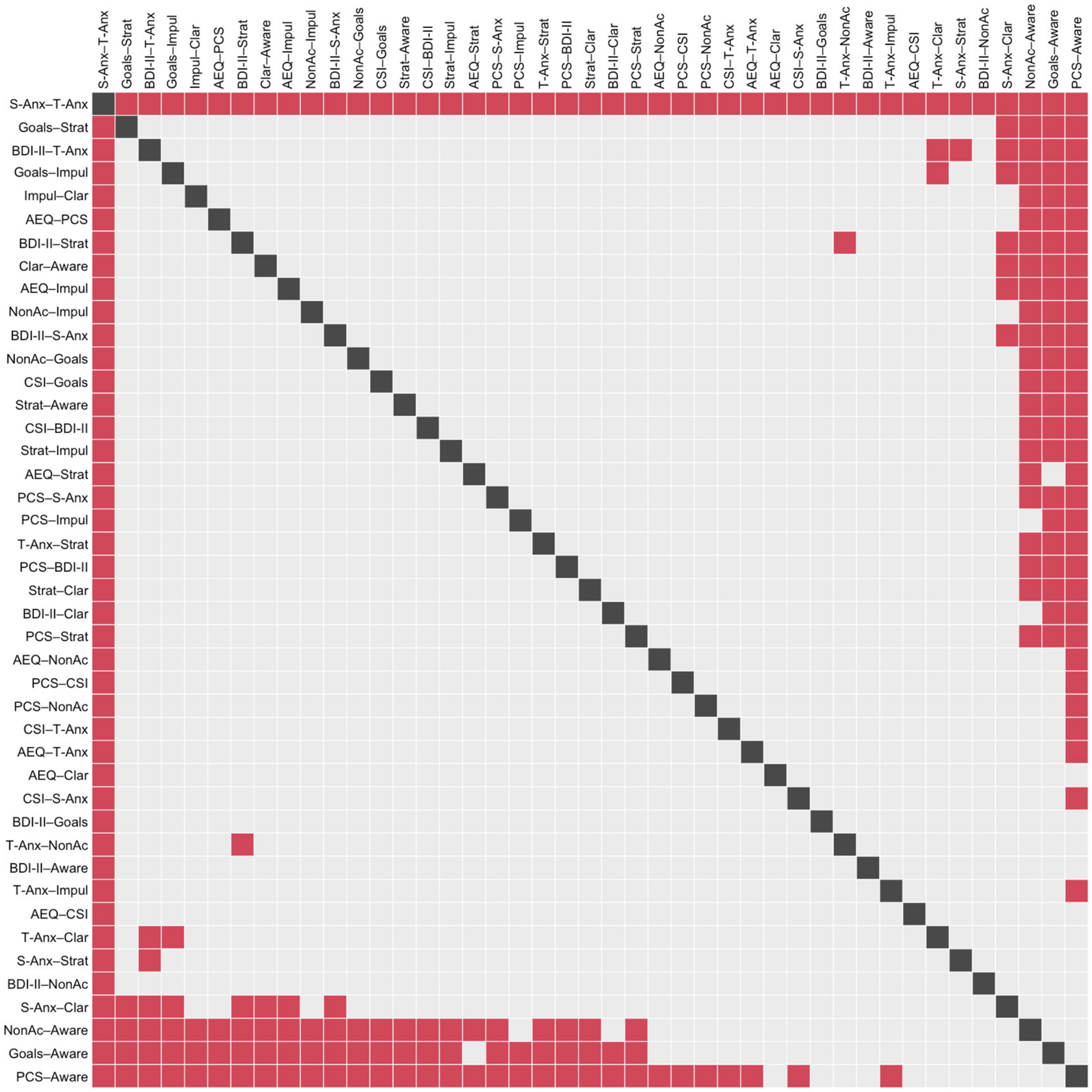

Figure S1b. Edge-difference test (5000 replications), restricted to the 43 non-zero edges and ordered by edge weight. Red cells indicate pairs of edges whose weights differed significantly; that is, whose bootstrapped 95% interval of the difference excluded zero; grey cells indicate pairs that did not differ. As shown, 126 of the 903 comparisons were significant. The intervals are pointwise and unadjusted for the number of comparisons; this is an exploratory analysis and the count of significant cells is not a family-wise controlled result. Abbreviations: AEQ—Emotional Allodynia Questionnaire; PCS—Pain Catastrophizing Scale; CSI—Central Sensitization Inventory; BDI-II—Beck Depression Inventory-II; S-Anx and T-Anx—STAI-Y1 (state) and STAI-Y2 (trait) anxiety; NonAc, Goals, Strat, Impul, Clar and Aware—the Non-Acceptance, Goals, Strategies, Impulse, Clarity and Awareness subscales of the DERS.

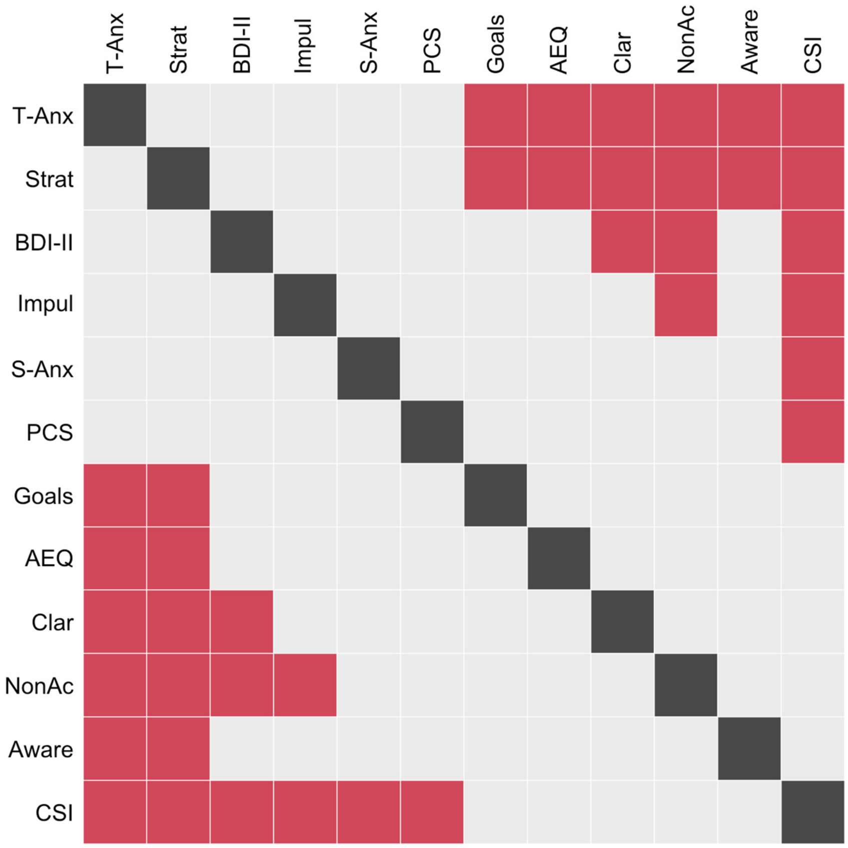

Figure S1c. Strength-difference test (5000 replications), with nodes ordered by strength centrality. Red cells indicate pairs of nodes whose strength differed significantly; 19 of the 66 comparisons were significant. Intervals follow the default percentile convention of the plotting routine (quantile type 7); the differenceTest function of the same package uses type 6 and returns 17. The intervals are pointwise and unadjusted for the number of comparisons; this is an exploratory analysis and the count of significant cells is not a family-wise controlled result. Abbreviations: AEQ—Emotional Allodynia Questionnaire; PCS—Pain Catastrophizing Scale; CSI—Central Sensitization Inventory; BDI-II—Beck Depression Inventory-II; S-Anx and T-Anx—STAI-Y1 (state) and STAI-Y2 (trait) anxiety; NonAc, Goals, Strat, Impul, Clar and Aware—the Non-Acceptance, Goals, Strategies, Impulse, Clarity and Awareness subscales of the DERS.

Table S8. Goldbricker redundancy analysis (n = 149). Node pairs whose correlation profiles with the remaining nodes did not differ significantly in more than 25% of comparisons, and which are therefore flagged as potentially redundant.

**Node pair**	**Proportion of significant differences**
PCS and AEQ	0.0
Impul and AEQ	0.0
T-Anx and BDI-II	0.1
Impul and Goals	0.1
S-Anx and AEQ	0.2
T-Anx and AEQ	0.2
Impul and PCS	0.2

S-Anx and BDI-II	0.2
Strat and BDI-II	0.2
Strat and T-Anx	0.2
Impul and T-Anx	0.2
Goals and NonAc	0.2

Note: AEQ—Emotional Allodynia Questionnaire; PCS—Pain Catastrophizing Scale; CSI—Central Sensitization Inventory; BDI-II—Beck Depression Inventory-II; S-Anx—STAI-Y1 (state anxiety); T-Anx—STAI-Y2 (trait anxiety); NonAc, Goals, Strat, Impul, Clar and Aware—the six DERS facets (Non-Acceptance, Goals, Strategies, Impulse, Clarity, Awareness).

Table S9. Partial Spearman correlations of the AEQ and of the PCS with the remaining nodes, each adjusted for the other (n = 149). The difference between the two measures was tested on difficulties in emotional clarity and on depression; for neither is it statistically significant. Here, STAI-Y1 denotes state anxiety and STAI-Y2 trait anxiety. A test for dependent correlations gave p = 0.212 for emotional clarity and p = 0.154 for depression, and a bootstrap of the difference between the reciprocal partial correlations included zero in both cases. The values below are the means of the bootstrap distribution, which differ slightly from the difference between the two coefficients in the observed sample: emotional clarity +0.168 (observed +0.170), 95% CI -0.104 to +0.443; depression -0.175 (observed -0.176), 95% CI -0.436 to +0.087.

**Node**	**AEQ adjusted for PCS**	**p**	**PCS adjusted for AEQ**	**p**
BDI-II	0.251	0.002	0.427	< 0.001
CSI	0.229	0.005	0.319	< 0.001
STAI-Y1	0.255	0.002	0.426	< 0.001
STAI-Y2	0.350	< 0.001	0.385	< 0.001
DERS Non-Acceptance	0.276	< 0.001	0.267	0.001
DERS Goals	0.257	0.002	0.253	0.002
DERS Strategies	0.377	< 0.001	0.362	< 0.001
DERS Impulse	0.405	< 0.001	0.335	< 0.001
DERS Clarity	0.313	< 0.001	0.142	0.084
DERS Awareness	0.085	0.304	-0.079	0.338

Note: each coefficient is the partial Spearman correlation of the row variable with the AEQ controlling for the PCS, or with the PCS controlling for the AEQ, and for nothing else. AEQ—Emotional Allodynia Questionnaire; PCS—Pain Catastrophizing Scale; CSI—Central Sensitization Inventory; BDI-II—Beck Depression Inventory-II; STAI-Y2—trait anxiety; DERS—Difficulties in Emotion Regulation Scale.

Table S10. Edge weights of the network re-estimated with threshold = TRUE, which enforces higher specificity at the cost of sensitivity. Fifteen of the 43 edges were retained; dashes denote edges set to zero (n = 149).

	**AEQ**	**PCS**	**CSI**	**BDI-II**	**S-Anx**	**T-Anx**	**NonAc**	**Goals**	**Strat**	**Impul**	**Clar**	**Aware**
AEQ	—	0.185	—	—	—	—	—	—	—	0.184	—	—
PCS	0.185	—	—	0.139	—	—	—	—	—	—	—	—
CSI	—	—	—	0.164	—	—	—	—	—	—	—	—
BDI-II	—	0.139	0.164	—	0.174	0.211	—	—	0.194	—	—	—
S-Anx	—	—	—	0.174	—	0.625	—	—	—	—	—	—
T-Anx	—	—	—	0.211	0.625	—	—	—	0.099	—	—	—
NonAc	—	—	—	—	—	—	—	—	—	0.192	—	—

Goals	—	—	—	—	—	—	—	—	0.237	0.213	—	—
Strat	—	—	—	0.194	—	0.099	—	0.237	—	0.162	—	0.218
Impul	0.184	—	—	—	—	—	0.192	0.213	0.162	—	0.225	—
Clar	—	—	—	—	—	—	—	—	—	0.225	—	—
Aware	—	—	—	—	—	—	—	—	0.218	—	—	—

Note: AEQ—Emotional Allodynia Questionnaire; PCS—Pain Catastrophizing Scale; CSI—Central Sensitization Inventory; BDI-II—Beck Depression Inventory-II; S-Anx—STAI-Y1 (state anxiety); T-Anx—STAI-Y2 (trait anxiety); NonAc, Goals, Strat, Impul, Clar and Aware—the six DERS facets (Non-Acceptance, Goals, Strategies, Impulse, Clarity, Awareness).

Table S11. Edge weights of the network re-estimated with the EBIC hyperparameter set to $\gamma = 0.25$, the value used by Oliva et al., instead of the more conservative $\gamma = 0.5$ adopted here (n = 149).

	AEQ	PCS	CSI	BDI-II	S-Anx	T-Anx	NonAc	Goals	Strat	Impul	Clar	Aware
AEQ	—	0.190	0.056	—	—	0.082	0.102	—	0.124	0.184	0.078	—
PCS	0.190	—	0.101	0.114	0.121	—	0.088	—	0.102	0.119	—	-0.121
CSI	0.056	0.101	—	0.146	0.067	0.084	—	0.163	—	—	—	—
BDI-II	—	0.114	0.146	—	0.180	0.208	0.021	0.067	0.185	—	0.112	0.059
S-Anx	—	0.121	0.067	0.180	—	0.583	—	—	0.030	—	0.003	—
T-Anx	0.082	—	0.084	0.208	0.583	—	0.060	—	0.116	0.058	0.030	—
NonAc	0.102	0.088	—	0.021	—	0.060	—	0.166	—	0.183	—	-0.068
Goals	—	—	0.163	0.067	—	—	0.166	—	0.223	0.193	—	-0.078
Strat	0.124	0.102	—	0.185	0.030	0.116	—	0.223	—	0.137	0.113	0.152
Impul	0.184	0.119	—	—	—	0.058	0.183	0.193	0.137	—	0.191	—
Clar	0.078	—	—	0.112	0.003	0.030	—	—	0.113	0.191	—	0.184
Aware	—	-0.121	—	0.059	—	—	-0.068	-0.078	0.152	—	0.184	—

Note: the network estimated with $\gamma = 0.25$ is identical to the one estimated with $\gamma = 0.5$ (43 non-zero edges). AEQ—Emotional Allodynia Questionnaire; PCS—Pain Catastrophizing Scale; CSI—Central Sensitization Inventory; BDI-II—Beck Depression Inventory-II; S-Anx—STAI-Y1 (state anxiety); T-Anx—STAI-Y2 (trait anxiety); NonAc, Goals, Strat, Impul, Clar and Aware—the six DERS facets (Non-Acceptance, Goals, Strategies, Impulse, Clarity, Awareness).

Table S12. Sensitivity analysis: edges of the AEQ under the nonparanormal transformation. The network was re-estimated after transforming all twelve variables with the nonparanormal transformation (huge::huge.npn), the procedure recommended for continuous non-normal data, and compared with the Spearman solution of the main analysis (n = 149; EBICglasso, $\gamma = 0.5$). Across all 66 node pairs, the two solutions correlated r = 0.986 and agreed on the presence or absence of an edge in 62 of 66 cases (43 non-zero edges under Spearman, 45 under the nonparanormal transformation; largest absolute difference in weight 0.057). The two strongest edges of the AEQ were the same and in the same order, and its edges with the BDI-II and with STAI-Y1 were exactly zero under both specifications. The four edges that differed in inclusion status were STAI-Y1–DERS Clarity (0.003 under Spearman, zero under the transformation), PCS–STAI-Y2 (zero, 0.022), AEQ–DERS Goals (zero, 0.021) and DERS Non-Acceptance–DERS Clarity (zero, 0.042); none reached an absolute weight of 0.05, and the only one involving the AEQ is its edge with DERS Goals, which Section 3.4 reports as zero. Dashes denote edges set to zero.

Node	Spearman	Nonparanormal	Difference
**PCS**	0.190	0.216	+0.026
**DERS Impulse**	0.184	0.193	+0.009
**DERS Strategies**	0.124	0.109	-0.015
**DERS Non-Acceptance**	0.102	0.105	+0.003
**STAI-Y2**	0.082	0.083	+0.001
**DERS Clarity**	0.078	0.053	-0.025

**CSI**	0.056	0.066	+0.010
**BDI-II**	—	—	0.000
**STAI-Y1**	—	—	0.000
**DERS Awareness**	—	—	0.000
**DERS Goals**	—	0.021	+0.021

Note: each value is the regularized partial correlation between the AEQ and the row variable, conditional on all ten remaining nodes. AEQ—Emotional Allodynia Questionnaire; PCS—Pain Catastrophizing Scale; CSI—Central Sensitization Inventory; BDI-II—Beck Depression Inventory-II; STAI-Y1—State-Trait Anxiety Inventory, state form; STAI-Y2— trait form; DERS— Difficulties in Emotion Regulation Scale.

Table S13. Exploratory factor structure of the twelve nodes (n = 149, Spearman correlation matrix). Parallel analysis against 1000 simulated datasets indicated three factors and one component. A single-factor solution accounted for 48.7% of the variance and reproduced the correlation matrix with a root mean square residual of 0.049, but its Tucker–Lewis index was 0.839 and seven of the 66 off-diagonal residuals exceeded 0.10 in absolute value (largest 0.215, DERS Clarity with DERS Awareness), so the matrix is not adequately described by one factor. Loadings are from an unrotated single-factor solution (principal axis) and are shown beside the strength centrality of the same node in the network of the main analysis. The two are correlated at r = 0.745 across the twelve nodes.

Node	Loading	Communality	Uniqueness	Strength
STAI-Y2	0.863	0.745	0.255	1.222
BDI-II	0.821	0.675	0.325	1.092
DERS Strategies	0.82	0.672	0.328	1.182
STAI-Y1	0.807	0.651	0.349	0.984
DERS Impulse	0.743	0.552	0.448	1.065
PCS	0.716	0.512	0.488	0.956
AEQ	0.699	0.488	0.512	0.815
DERS Goals	0.667	0.444	0.556	0.889
CSI	0.635	0.403	0.597	0.618
DERS Non-Acceptance	0.584	0.341	0.659	0.688
DERS Clarity	0.583	0.34	0.66	0.711
DERS Awareness	0.15	0.023	0.977	0.663

Note: loadings are on the single common factor. Strength is the sum of the absolute weights of the node's edges in the network of the main analysis (Table S3). AEQ— Emotional Allodynia Questionnaire; PCS— Pain Catastrophizing Scale; CSI— Central Sensitization Inventory; BDI-II— Beck Depression Inventory-II; STAI-Y1/Y2— State–Trait Anxiety Inventory, state and trait forms; DERS—Difficulties in Emotion Regulation Scale.

Table S14. Sensitivity to node granularity: edges of the AEQ in a network in which the Pain Catastrophizing Scale is entered as its three subscales rather than as a total score (fourteen nodes; n = 149; EBICglasso, γ = 0.5, Spearman). Because the proportions of Table S5 depend on how many nodes each domain contains, this analysis enlarges the pain domain from two nodes to four. The shares directed by the AEQ were 33.6% at the pain domain, 9.6% at depression and anxiety and 56.9% at emotion regulation, against 30.2%, 10.0% and 59.8% in the twelve-node network, and its edge with the BDI-II remained exactly zero. Of the three catastrophizing subscales, the edge with Magnification was the largest (0.171), that with Helplessness was weak (0.062) and that with Rumination was zero. These three edges were re-estimated in 5000 non-parametric bootstrap replications, all of which converged. The Magnification edge was non-zero in 96.7% of them (95% percentile interval 0.000 to 0.302), the Helplessness edge in 74.4% (0.000 to 0.185) and the Rumination edge in 42.1%

(0.000 to 0.135); the percentile interval of the difference between the Magnification and the Rumination edge was -0.047 to +0.295 and includes zero. The ordering of the three subscales is therefore not established at this sample size and is reported as exploratory.

Node	Domain	Edge with the AEQ
PCS Rumination	Pain	0
PCS Magnification	Pain	0.171
PCS Helplessness	Pain	0.062
CSI	Pain	0.042
BDI-II	Depression and anxiety	0
STAI-Y1	Depression and anxiety	0
STAI-Y2	Depression and anxiety	0.078
DERS Non-Acceptance	Emotion regulation	0.107
DERS Goals	Emotion regulation	0
DERS Strategies	Emotion regulation	0.108
DERS Impulse	Emotion regulation	0.177
DERS Clarity	Emotion regulation	0.074
DERS Awareness	Emotion regulation	0

Note: PCS subscales were scored with the original item allocation (Rumination, items 8–11; Magnification, items 6, 7 and 13; Helplessness, items 1–5 and 12), and the sum of the thirteen items reproduced the stored total score for every participant. AEQ— Emotional Allodynia Questionnaire; PCS— Pain Catastrophizing Scale; CSI— Central Sensitization Inventory; BDI-II— Beck Depression Inventory-II; STAI-Y1/Y2— State–Trait Anxiety Inventory, state and trait forms; DERS— Difficulties in Emotion Regulation Scale.

Table S15. Node predictability re-estimated after removing state anxiety from the model (n = 149; mixed graphical model, all nodes Gaussian, EBIC with $\gamma = 0.5$). The two forms of the STAI correlate at $\varrho = 0.869$, so each predicts the other almost by construction, and the predictability reported for them in Table S3 is inflated by that redundancy. With only one anxiety scale in the model, the predictability of trait anxiety falls from 0.693 to 0.469, while that of the AEQ is unchanged at 0.402. The contrast discussed in Section 4.2 is therefore smaller than the twelve-node values suggest.

Node	R^2 (twelve nodes)	R^2 (without STAI-Y1)
AEQ	0.402	0.402
PCS	0.436	0.409
CSI	0.415	0.412
BDI-II	0.526	0.647
STAI-Y2	0.693	0.469
DERS Non-Acceptance	0.197	0.197
DERS Goals	0.496	0.496
DERS Strategies	0.467	0.467
DERS Impulse	0.574	0.574
DERS Clarity	0.405	0.406
DERS Awareness	0.058	0.058

Note: R^2 is the proportion of variance in each node explained by its neighbours in the mixed graphical model, which estimates its own nodewise neighbourhoods and is not identical to the displayed network. AEQ— Emotional Allodynia Questionnaire; PCS— Pain Catastrophizing Scale; CSI— Central Sensitization Inventory; BDI-II— Beck Depression Inventory-II; STAI-Y1/Y2— State–Trait Anxiety Inventory, state and trait forms; DERS— Difficulties in Emotion Regulation Scale.

Table S16. Sensitivity to node granularity in both directions: the proportion of the AEQ's total connectivity directed at each domain under four representations of the same data.

Specification	Nodes in the network	Pain	Depression and anxiety	Emotion regulation
Facet-level (primary; Section 3.3)	12	30.2	10.0	59.8
PCS entered as three subscales (Table S14)	14	33.6	9.6	56.9
DERS entered as a total score	7	45.0 (17.0–66.2)	13.8 (0.7–40.8)	41.1 (15.8–61.5)
Shares normalized per available node	12	53.2 (16.7–73.2)	11.7 (0.0–34.1)	35.1 (16.8–64.6)

Note: shares are computed from absolute edge weights, as in Table S5, and sum to 100%. The number of nodes available in each domain is two, three and six, respectively, in the facet-level network; four, three and six with the PCS entered as three subscales; and two, three and one with the DERS entered as a total score. Bootstrap percentile intervals (2.5th–97.5th) are given in parentheses. Those for the normalized shares were recomputed on the frozen bootstrap distribution of Figures S1a–c, as in Table S5. Those for the instrument-level network come from a separate non-parametric bootstrap of 5000 replications of that network (5000/5000 converged) and are not comparable with the intervals of Tables S4, S5 or S7, which are computed on different networks. In the instrument-level network, the AEQ retained edges with the DERS total score (0.282; non-zero in 100% of replications), the PCS (0.243; 99.8%), trait anxiety (0.095; 90.8%) and the CSI (0.066; 78.3%); its edge with the BDI-II was zero, selected in 42.6% of replications, as was its edge with state anxiety, selected in 37.4%. n = 149 throughout.

Table S17. Attenuation of the association between the AEQ and the BDI-II across a nested sequence of adjustment sets (n = 149).

Adjustment set	Variables held constant	Partial ρ	p	Change from previous step
Zero-order	none	0.502	< 0.001	—
Adjusted for pain catastrophizing	PCS	0.251	0.002	−0.251
Further adjusted for emotion dysregulation	PCS + six DERS facets	0.044	0.607	−0.207
Further adjusted for anxiety	PCS + DERS + STAI-Y1 + STAI-Y2	−0.015	0.857	−0.059
Full model	PCS + DERS + STAI-Y1 + STAI-Y2 + CSI	−0.033	0.703	−0.018

Note: Partial Spearman correlations, computed from the inverse of the Spearman correlation matrix of the variables in each set; p-values are nominal and uncorrected. The sequence is descriptive and does not identify mediation: the variables held constant may act as confounders, mediators, parallel indicators or colliders, and a cross-sectional model cannot distinguish these roles. Almost all of the attenuation occurs in the first two steps, pain catastrophizing halving the association and the six emotion-dysregulation facets absorbing most of the remainder; the two anxiety scales and the CSI together change it by less than 0.08.

Table S18. Evidence bearing on the absence of a conditional association between the AEQ and the BDI-II, none of which relies on LASSO selection (n = 149).

Quantity	Value

Zero-order Spearman correlation	0.502 ($p < 0.001$)
Unpenalized partial Spearman correlation, full model	−0.033 ($p = 0.703$)
95% bootstrap percentile interval of that partial correlation	−0.229 to 0.162
90% bootstrap percentile interval of that partial correlation	−0.198 to 0.130
Upper one-sided 95th percentile for a positive residual association	0.130
Change in BIC when the BDI-II is added to the model	+4.845
Approximate BIC evidence ratio favouring the model without the BDI-II	11.3 to 1
Increase in R^2 attributable to the BDI-II	0.00054
Repetitions in which adding the BDI-II improved cross-validated RMSE	0 of 100
Equivalence (TOST) at a margin of $\|\rho\| < 0.15$	not reached ($p = 0.084$)
Equivalence (TOST) at a margin of $\|\rho\| < 0.20$	reached ($p = 0.024$)

Note: Bootstrap intervals are percentile intervals from 5000 non-parametric replications of the unpenalized partial correlation (5000/5000 valid); they are a third bootstrap distribution and are not comparable with the intervals of Tables S4, S5, S7 or S16. BIC and R^2 come from rank-based linear models of the AEQ on the remaining nodes, fitted with and without the BDI-II; cross-validation is ten-fold, repeated 100 times. The BIC evidence ratio is a post hoc approximation computed from the difference in BIC and is not a Bayes factor from a pre-specified Bayesian model. No minimum relevant effect size was specified in advance, so the equivalence margins are reported as a range rather than tested against a single prespecified value; the analysis therefore supports omitting the BDI-II relative to retaining it, and does not establish equivalence to zero.

Table S19. Characteristics of the three reports from the PEARL cohort.

Characteristic	Corriero et al., J Pain Res 2026	Corriero et al., Front Psychol 2026	Present report
**Sample size**	107	136	149
**Recruitment period and data cut-off**	September 2025 to January 2026	From September 2025; cut-off by May 2026	September 2025 to July 2026
**Participants contained in the present sample**	All 107	All 136	—
**Variables examined**	AEQ, CSI, PCS, BDI-II, STAI-Y1, STAI-Y2 and DERS total score; clinical and treatment characteristics	AEQ and the six DERS facets, with BDI-II, STAI-Y2, PCS and CSI as covariates	AEQ, PCS, CSI, BDI-II, STAI-Y1, STAI-Y2 and the six DERS facets
**Primary research question**	Preliminary psychometric validation of the AEQ and exploratory AEQ–CSI phenotyping	Which DERS facets are associated with the AEQ beyond depressive symptoms, trait anxiety, pain catastrophizing and central sensitization	Position of the AEQ in a network of pain-related, affective and emotion-regulation measures; its conditional association with depressive symptoms; its distinction from pain catastrophizing
**Statistical analyses**	Cronbach's α; exploratory and confirmatory factor analysis in the same sample; Spearman correlations;	Zero-order and partial Spearman correlations; hierarchical linear regression	Regularized partial correlation network (EBICglasso, Spearman); bootstrap accuracy and case-

	AEQ–DERS cross-tabulation and Fornell–Larcker criterion; principal component analysis; median-split phenotypes with Kruskal–Wallis tests	with facets entered singly and simultaneously	dropping stability; bridge centrality and domain shares; predictability; redundancy analysis; unpenalized partial correlations, BIC comparison and cross-validation; sensitivity analyses
**Estimates re-estimated in the present report**	Zero-order correlations of the AEQ with the CSI, PCS, BDI-II, STAI-Y1 and STAI-Y2, and among those instruments	Zero-order correlations of the AEQ with the six DERS facets; partial correlations under a different adjustment set	—

Note: the three reports are successive analyses of one continuously recruited single-centre cohort (PEARL study, Pain Clinic of Policlinico Hospital, Bari), and the participants of both earlier reports are contained in the present sample. Estimates that appear in more than one report are re-estimations in an enlarged sample, not independent replications. AEQ— Emotional Allodynia Questionnaire; BDI-II— Beck Depression Inventory-II; CSI— Central Sensitization Inventory; DERS— Difficulties in Emotion Regulation Scale; PCS— Pain Catastrophizing Scale; STAI-Y1/Y2— State–Trait Anxiety Inventory, state and trait forms.

Table S20. Association between the AEQ and the BDI-II adjusted for, and stratified by, documented depressive disorder and current antidepressant use (n = 149).

**Model or stratum**	**n**	**Zero-order ϱ**	**Partial ϱ (p)**	**95% percentile interval**
Full cohort, adjusted for the other ten nodes	149	0.502	−0.033 (0.703)	−0.228 to 0.166
Full cohort, + documented depressive disorder	149	0.502	−0.034 (0.694)	−0.234 to 0.168
Full cohort, + current antidepressant use	149	0.502	−0.034 (0.696)	−0.237 to 0.166
Full cohort, + both	149	0.502	−0.039 (0.647)	−0.246 to 0.164
Documented depressive disorder	68	0.385	−0.096 (0.473)	−0.415 to 0.255
No documented depressive disorder	81	0.567	−0.111 (0.358)	−0.339 to 0.118
Current antidepressant use	58	0.459	0.041 (0.782)	−0.300 to 0.389
No current antidepressant use	91	0.530	−0.068 (0.547)	−0.325 to 0.176

Note: partial Spearman correlations between the AEQ and the BDI-II. In the full cohort they are adjusted for the other ten nodes and, where stated, for documented depressive disorder and current antidepressant use entered as binary variables; within strata they are adjusted for the other ten nodes. Intervals are percentile intervals from 5000 non-parametric replications (5000/5000 valid in every row), drawn with a seed different from that of Table S18, which explains the slightly different interval in the first row; they are not comparable with the bootstrap distributions of other tables. p-values are nominal and uncorrected. Thirty-three patients had both a documented depressive disorder and a current antidepressant, 35 had the disorder only, 25 had the antidepressant only and 56 had neither. In rank-based linear models of the AEQ on the remaining eleven nodes, the interaction between the BDI-II and each binary variable, entered separately, gave no evidence of modification (depressive disorder, p = 0.299; antidepressant use, p = 0.393); this is not evidence of equivalence across strata, whose intervals are wide. Documented depressive disorder and antidepressant use are coded as in Table 1 of the main text; a code of zero means that no diagnosis or drug was recorded.

Table S21. Agreement between the predictors of the nodewise predictability models and the edges of the network (n = 149).

Node	Edges in the network	Predictors retained by the nodewise model	R^2
**AEQ**	7	PCS, STAI-Y2, DERS Strategies, DERS Impulse	0.402
**PCS**	8	AEQ, BDI-II, STAI-Y1, DERS Strategies, DERS Impulse	0.436
**CSI**	6	AEQ, PCS, BDI-II, STAI-Y1, STAI-Y2, DERS Goals	0.415
**BDI-II**	9	STAI-Y1, STAI-Y2, DERS Strategies	0.526
**STAI-Y1**	6	STAI-Y2	0.668
**STAI-Y2**	8	BDI-II, STAI-Y1	0.693
**DERS Non-Acceptance**	7	DERS Goals, DERS Impulse	0.197
**DERS Goals**	6	CSI, BDI-II, DERS Non-Acceptance, DERS Strategies, DERS Impulse	0.496
**DERS Strategies**	9	BDI-II, STAI-Y2, DERS Goals, DERS Impulse	0.467
**DERS Impulse**	7	AEQ, PCS, STAI-Y2, DERS Non-Acceptance, DERS Goals, DERS Strategies, DERS Clarity	0.574
**DERS Clarity**	7	AEQ, BDI-II, DERS Strategies, DERS Impulse, DERS Awareness	0.405
**DERS Awareness**	6	DERS Clarity	0.058

Note: Edges in the network are the non-zero edges of the regularized partial correlation network of the main analysis (43 in total). Predictors are the non-zero coefficients of the nodewise regressions of the mixed graphical model used for predictability (all nodes Gaussian, EBIC with $\gamma = 0.5$, same seed as Table S3), which are the coefficients from which R^2 is computed. All 45 retained predictors are also edges of the network. Combined across nodes with the AND rule, the mixed graphical model retains 14 edges, all of them among the 43 edges of the network (Jaccard index 0.326); the remaining 29 network edges are not retained. R^2 is computed on the same data used for estimation and describes in-sample fit, not out-of-sample prediction.
